# Spatial Metabolomics Reveals the Multifaceted Nature of Lamprey Buccal Gland and Its Diverse Mechanisms for Blood-Feeding

**DOI:** 10.1038/s42003-023-05250-x

**Published:** 2023-08-28

**Authors:** Meng Gou, Xuyuan Duan, Jun Li, Yaocen Wang, Qingwei Li, Yue Pang, Yonghui Dong

**Affiliations:** 1 College of Life Science, Liaoning Normal University, Dalian, 116081 China; 2 Lamprey Research Center, Liaoning Normal University, Dalian, 116081 China; 3 Life Sciences Core Facilities, Weizmann Institute of Science, Rehovot, 7610001 Israel

**Keywords:** Metabolomics, Small molecules

## Abstract

Lampreys are blood-sucking vampires in marine environments. From a survival perspective, it is expected that the lamprey buccal gland exhibits a repository of pharmacologically active components to modulate the host’s homeostasis, inflammatory and immune responses. By analyzing the metabolic profiles of 14 different lamprey tissues, we show that two groups of metabolites in the buccal gland of lampreys, prostaglandins and the kynurenine pathway metabolites, can be injected into the host fish to assist lamprey blood feeding. Prostaglandins are well-known blood-sucking-associated metabolites that act as vasodilators and anticoagulants to maintain vascular homeostasis and are involved in inflammatory responses. The vasomotor reactivity test on catfish aortic ring showed that kynurenine can also relax the blood vessels of the host fish, thus improving the blood flow of the host fish at the bite site. Finally, a lamprey spatial metabolomics database (https://www.lampreydb.com) was constructed to assist studies using lampreys as animal model.

## Abstract (web summary)

A spatial metabolomics study of the lampreys leads to the identification of the kynurenine pathway metabolites and prostaglandins in their buccal gland secretions (saliva), which are likely involved in promoting blood flow when feeding on host fishes.

## Introduction

Lampreys, together with hagfishes, are the only extant lineages of jawless fish^1,2^. Accumulating fossil evidence has demonstrated that lampreys in the Devonian period were already almost identical to the modern adult lampreys, with well-developed oral disc, annular cartilages, and circumoral teeth^3–6^, suggesting the evolutionary long-term stability of lampreys.

Lampreys are aquatic, eel-shaped animals. Some species live in freshwater for their entire lives such as the Korean lamprey (*Eudontomyzon morii*), while others, including the sea lamprey (*Petromyzon marinus*) and the Arctic lamprey (*Lethenteron camtschaticum*), usually migrate to the sea to feed^7^. The life cycle of all lampreys begins with a freshwater larval phase (also called ammocoetes), in which the larval lampreys live burrowed in the substrate of streams as filter feeders. After about 3–7 years or more^5,8^, all lampreys complete metamorphosis into juvenile lampreys, with their characteristic oral disc and dagger-like tongue. In parasitic species of lampreys, the oral disc and dagger-like tongue is used to attach to and pierce the hide of fishes to allow them to ingest blood^9^. After a year or more, the juvenile lampreys become sexually mature adults which no longer feed. By contrast, the non-parasitic lampreys do not feed after the completion of metamorphosis^10–12^. In the last stage, the adult lampreys return to freshwater to spawn and die^7,13^.

Forty lamprey species are currently recognized for the extant lampreys, of which 18 species are parasitic^14^. Almost all blood-sucking animals are invertebrates, such as fleas, ticks, leeches, and mosquitoes, and lampreys are one of the only a few groups of vertebrate ectoparasites^15^. Parasitic lampreys usually attach themselves to the body surface of the host through their sucker-like oral disc, rasp a hole in the skin with a tongue-like piston tipped with denticles that form the cutting edges, and suck the blood of the host for days. As such, parasitic lampreys must suppress the immune response (that can lead to itching or pain and thus trigger defensive behavior on their hosts), nociceptive response (that can initiate host defense behavior), and hemostasis (the vertebrate mechanisms that prevent blood loss) of the host to ensure successful and long-term blood feeding. Extensive studies have revealed that the lamprey buccal gland secretes various proteins that function as anticoagulants, ion channel blockers, and immune suppressors^7,15,16^. However, metabolites (small molecules which act as intermediates or end products of cellular metabolism) in the buccal gland secretes have never been explored in detail. Given their unique phylogenetic position and status as one of the few groups of vertebrate ectoparasites, lampreys are expected to have developed distinct metabolites specifically adapted for blood-feeding and parasitism. Detecting and identifying these metabolites will improve our understanding in how lampreys ingest blood and provide new insights into the development of effective drugs in anti-inflammation and pain-relief. To this end, we have performed a spatial metabolomics analysis of 14 different lamprey tissues. The lamprey buccal gland was particularly investigated due to the reason that it is a blood-sucking organ, and that an unexpected rich and unique metabolic profile was detected in buccal gland. Finally, we have constructed a lamprey spatial metabolomics database to facilitate studies in biochemistry, clinical chemistry, natural product discovery, medicine, and metabolomics using lampreys as a model animal.

## Results

### Tissue-wide spatial metabolomics of lamprey

**Fig. 1:**
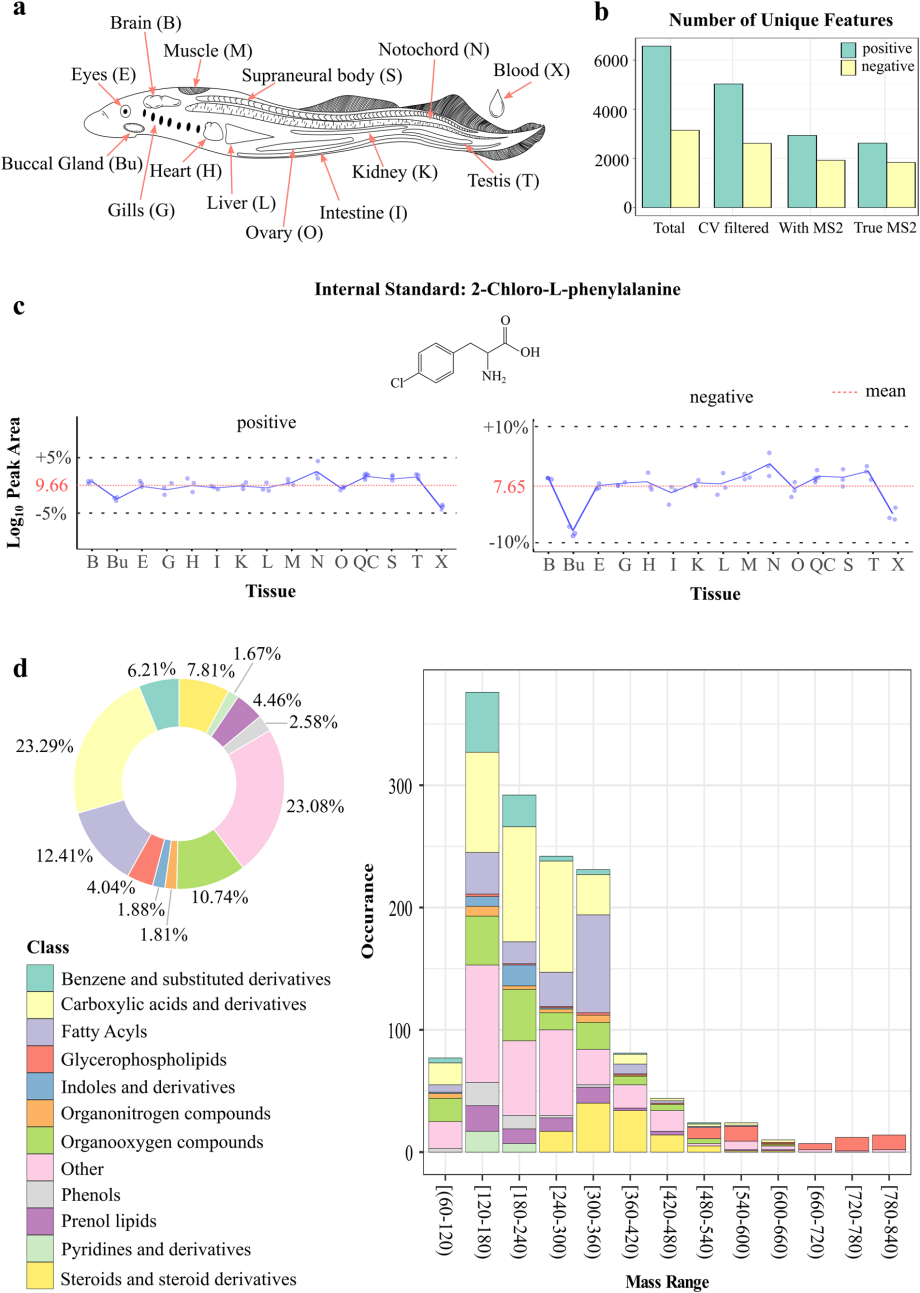
Overview of spatial metabolomics of lampreys. **a** Anatomical illustration of the 14 lamprey tissues subjected to metabolomics analysis. Each tissue has a unique abbreviation and is kept consistent throughout the figures. **b** Bar plot showing the number of unique mass features before and after data cleaning at positive and negative ion modes. **c** Line plots showing the log_10_ transformed peak area variations of internal standard, 4-chloro-L-phenylalanine, detected at positive and negative ion modes, respectively (*n* = 3 for each tissue). The red dashed line represents the mean peak areas across all the samples, and gray dashed lines are mean peak area ±5% of the mean peak area. **d** Summary of the total identified metabolites in different lamprey tissues. Pi chart showing the percentage of identified metabolites belonging to different chemical classes. Histogram showing the distribution of chemical classes along the mass range. Each chemical class is represented by a unique color code.

The Arctic lamprey (*Lethenteron camtschaticum*) were collected during their adult, spawning migration phase when they are no longer feeding. Fourteen lamprey tissues, i.e., heart, liver, kidney, brain, supraneural body, muscle, intestine, gill, eye, testis, ovary, buccal gland, blood, and notochord, were carefully dissected, and subjected to untargeted metabolomics using liquid chromatography mass spectrometry (LCMS) (Fig. 1a). The raw data quality and inter-batch variation were assessed on 5 pooled quality control (QC) samples obtained at both positive and negative ion modes (Supplementary Data 1 and 2). In addition, an internal standard (IS), 2-chloro-L-phenylalanine, was used for rapid inter-batch variation evaluation, and the result confirmed a good reproducibility within the samples (Fig. 1c).

In total, 6568 and 3143 unique features were detected in positive and negative ion modes, respectively (Fig. 1b). A data cleaning was then performed to remove unstable mass features without MS2 spectrum, and features with wrong MS2 spectrum (i.e., wrong precursor ion was selected for fragmentation). The resulting 2621 (positive ion mode) and 1835 (negative ion mode) mass features were left for subsequent metabolite identification and statistical analysis (Fig. 1b). Tentative and putative metabolite annotations were performed based on accurate mass measurements, isotope distribution similarity, and manual assessment of fragmentation spectrum matching (when applicable) against our in-house database (~3600 standards), and mzCloud (https://www.mzcloud.org), the Human Metabolome Database (https://hmdb.ca)^17^ and Lipid Map (https://www.lipidmaps.org)^18^, and all public MS-DIAL databases^19,20^. This step led to the detection of a large diverse of metabolite classes in lamprey; among them, carboxylic acid and its derivatives were the most abundant class (Fig. 1d).

### Buccal gland is a metabolic outlier tissue

**Fig. 2:**
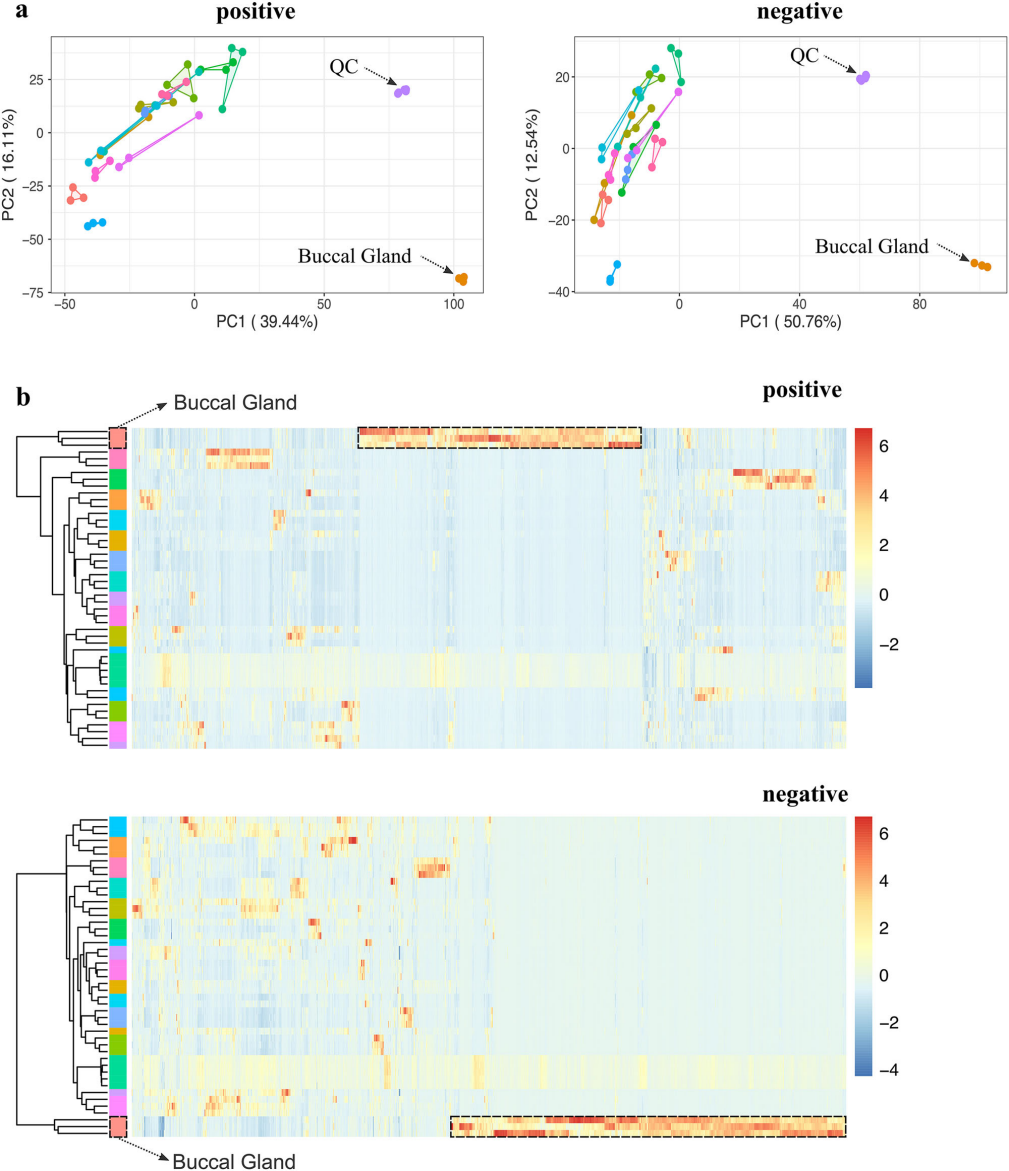
Principal component analysis (PCA) of the metabolic profiles of 14 different lamprey tissues. **a** PCA score plots of the metabolic profiles acquired at positive and negative ion modes, respectively (*n* = 3 for each tissue except that *n* = 5 for QC samples). **b** Hierarchical clustering heatmaps of the metabolic profiles acquired at positive and negative ion modes, respectively (*n* = 3 for each tissue except that *n* = 5 for QC samples). Each row represents a sample, and each column represents a metabolite. Each colored cell (red higher, blue lower) on the map corresponds to a z-score normalized mass feature.

Principal component analysis (PCA) was applied for initial examination of the metabolic profiles of different lamprey tissues. The PCA score plots from both positive and negative ion mode data revealed that the buccal gland was far separated from all the other tissues (Fig. 2a), suggesting that buccal gland had a distinct metabolic profile. Hierarchical clustering heatmap showed that one-third of the mass features were highly abundant in buccal gland in positive ion mode (Fig. 2b), and more than half of the features were enriched in buccal gland in negative ion mode (Fig. 2b). Further statistical analysis showed a significant accumulation of metabolites in buccal gland. For instance, 127 and 182 mass features were found over 1000-times higher in buccal gland compared to all the other 13 tissues (FDR-adjusted *p*-value < 0.001) in positive and negative ion modes, respectively. It is worth noting that it is typically not straightforward to directly compare the metabolic profiles of different tissues due to the differences in tissue-specific matrix effects^21,22^. However, the equal detection of internal standard (IS, 4-Chloro-L-phenylalanine) in different tissue groups may indicate that the tissue-specific matrix effects differences are not significant in our study (Fig. 1c). Nevertheless, our objective here is not to identify biomarkers to distinguish the buccal gland from any other lamprey tissues. Instead, here we would like to detect lamprey buccal gland-specific metabolites.

Parasitic lampreys are known to secrete proteins that possess anticoagulant and vasodilator activities from the buccal glands during feeding on their hosts^13^. Intensive studies have been performed to detect, identify and study the functions of those proteins^7,13,15,16^. In contrast, there have been surprisingly few studies on small molecules in lamprey buccal glands^23^. Considering the rich and unique metabolic profiles of the buccal gland and its important biological function to promote blood flow from the host to the parasite, we have therefore focused on the buccal gland in the subsequent studies to investigate the lamprey blood-sucking mechanism at the functional metabolic level.

### Tryptophan-kynurenine pathway metabolites and prostaglandins are highly accumulated in the buccal gland

**Fig. 3:**
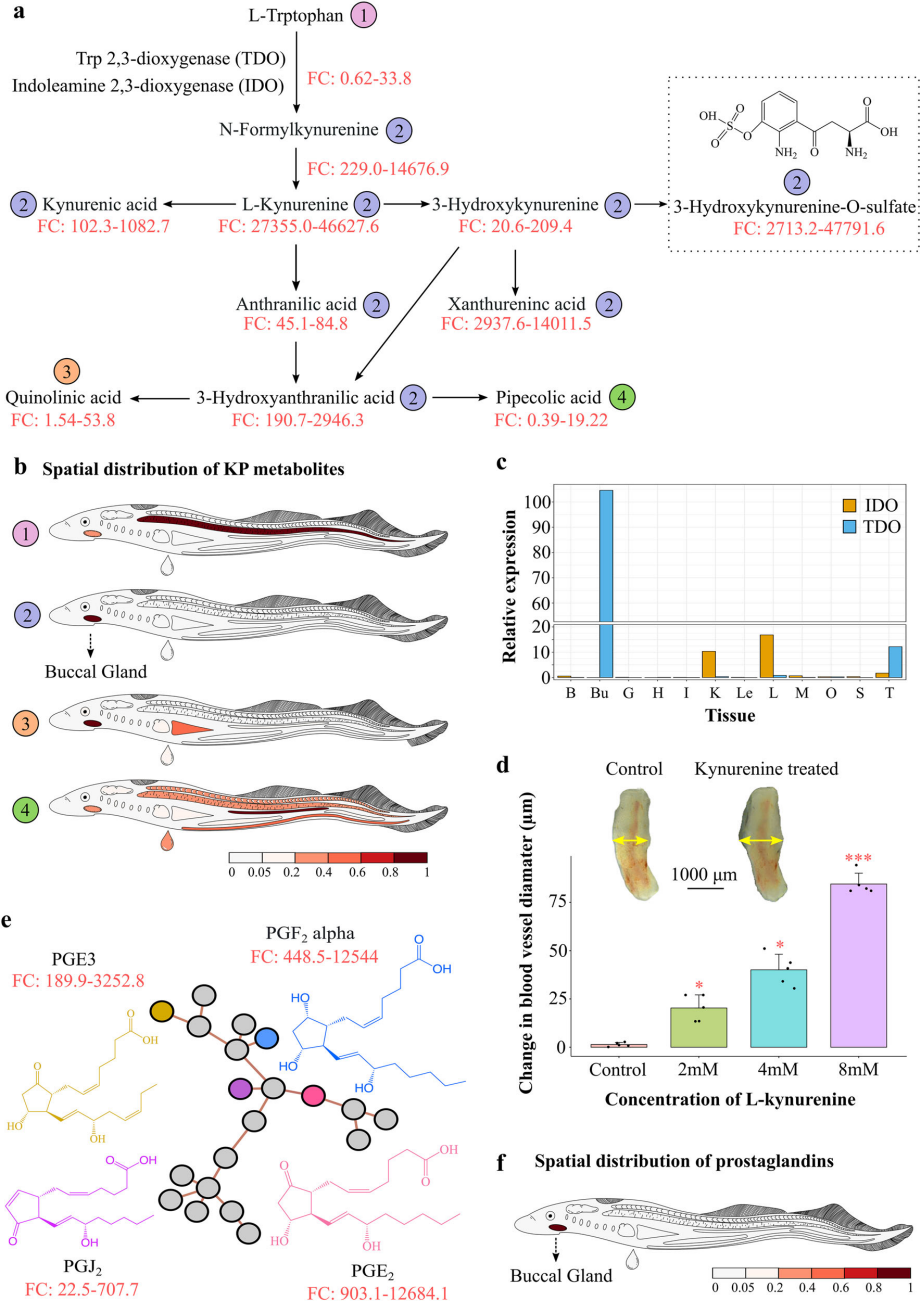
Kynurenine pathway metabolites and prostaglandins detected in lamprey buccal gland. **a** Schematic representation of the kynurenine pathway (KP). The fold change (FC) value was calculated for each metabolite in the pathway by dividing the peak area of that metabolite detected in the buccal gland by that in all the other lamprey tissues (QC samples were excluded). The FC value range was then displayed underneath each metabolite name. The chemical structure for 3-hydroxykynurenine-O-sulfate, a lamprey-unique KP metabolite was shown in the pathway. **b** Four unique spatial distribution patterns were detected for KP metabolites. L-tryptophan was accumulated in the notochord (circled in purple); quinolinic acid was mostly found in lamprey buccal gland and liver (circled in orange); pipecolic acid was more abundant in kidney (circled in green); and all the rest KP metabolites were exclusively accumulated in lamprey buccal gland (circled in blue). **c** the relative expression of tryptophan 2,3-dioxygenase (TDO) and indoleamine 2,3-dioxygenase (IDO) in different lamprey tissues. The tissue abbreviations are the same as shown in Fig. 1a. **d** Bar plot showing the changes in catfish blood vessel diameter upon treatments of different concentrations of kynurenine. The data are shown as the mean ± SD (*n* = 3). The insert representative figures are the optical images of catfish blood vessels before and after kynurenine treatment. Asterisks denote significant differences (*p < 0.05; ***p < 0.001; Wilcoxon signed-rank test) between kynurenine**-**treated and PBS treated (control) blood vessels. **e** A sub molecular network of prostaglandins was detected in lamprey buccal gland. Chemical structures of the 4 identified prostaglandins were displayed. The fold change (FC) value range of each prostaglandin was calculated in the same way as described in Fig. 3a. Gray node represents unidentified mass peaks or in-source fragments of the identified metabolites. **f** A representative anatomical heatmap showed that all the 4 prostaglandins were exclusively localized in lamprey buccal gland.

In total, more than 1500 mass features were found highly abundant in lamprey buccal gland (FC > = 10 and FDR-adjusted *p*-value < 0.05). Among them, 272 were tentatively identified and they belonged to over 30 different chemical classes, such as fatty acyls, steroids, and steroid derivatives. These buccal gland-specific mass features are perfect candidates for screening blood-sucking associated metabolites. Notably, a complete kynurenine pathway (KP) was detected in the buccal gland (Fig. 3a). The MS/MS spectrum of each KP pathway metabolite, annotation of their major fragments, and head-to-tail library match plots are shown in Supplementary Figs. 1–6. As clearly shown in the anatomical heatmap, most of the KP metabolites were exclusively accumulated in buccal gland (Fig. 3a, b). For instance, N-formylkynurenine was found between 229.0 and 14676.9 times higher in buccal gland compared to all the other 13 tissues, and kynurenine was between 27355 and 46627.6 times higher in buccal gland (Fig. 3a). In addition, a lamprey buccal gland-specific KP pathway metabolite, namely 3-hydroxykynurenine-O-sulfate^23^, was also identified with its fold change values ranging from 2713.2 to 47791.6 in buccal gland compared to other tissues (Fig. 3a). Although its function is still unclear, the detection of 3-hydroxykynurenine-O-sulfate in other blood-sucking insects, such as *Rhodnius prolixus*^24^, suggests that it might be a blood-feeding related metabolite. The KP is rate-limited by its first enzymes, tryptophan 2,3-dioxygenase (TDO) and indoleamine 2,3-dioxygenase (IDO), which convert tryptophan into N-formylkynurenine^25,26^ (Fig. 3a). The expression levels of the two major genes were studied by real-time quantitative PCR (qPCR), and the result showed that *TDO* was highly expressed in the buccal gland while *IDO* was mostly in the liver (Fig. 3c).

The KP has received increasing attention nowadays due to its emerging associations with inflammation, the immune system, and neurological conditions^25,26^. These unique roles of KP are potentially associated with lamprey blood-sucking. In addition, the report of 3-hydroxykynurenine-O-sulfate in other blood-sucking species makes KP even more attractive in investigating their roles in lamprey blood feeding. L-Kynurenine, in particular, has been reported to decrease vascular resistance and improve blood flow by relaxing blood vessels^27,28^. We, therefore, asked if L-kynurenine could also relax the blood vessels of the host fish. As such, we performed a vasomotor reactivity test on the catfish (*Silurus asotus*) aortic ring. As shown in Fig. 3d, compared to the control group (PBS-treated), the blood vessel diameter significantly increased 15 min after the addition of L-kynurenine, and the degree of increase appeared to be associated with the concentrations of L-kynurenine (Fig. 3d).

Apart from KP metabolites, four prostaglandins (PG), namely PGJ_2_, PGF_2_alpha, PGE_2_, and PGE_3_, were identified in the buccal gland using molecular networking analysis (Fig. 3e). The MS/MS spectrum of each prostaglandin, annotation of their major fragments, and head-to-tail library match plots are shown in Supplementary Figs. 7–10. Anatomical heatmap showed that all the 4 prostaglandins were exclusively accumulated in lamprey buccal gland (Fig. 3f). For instance, PGE_2_ was found between 903.1 and 12684.1 times higher in the buccal gland compared to all the other tissues (Fig. 3e).

### Tryptophan-kynurenine pathway metabolites and prostaglandins can be transferred from lampreys to their host fish

During blood-feeding, parasitic juvenile lampreys attach to the host fish by their oral disks, penetrate the skin through the action of their toothed tongue-like pistons, create a feeding niche at the bite site, and then start feeding^29^. On the other side, host defenses will attempt to counterattack and stop lamprey feeding at the bite site.

**Fig. 4:**
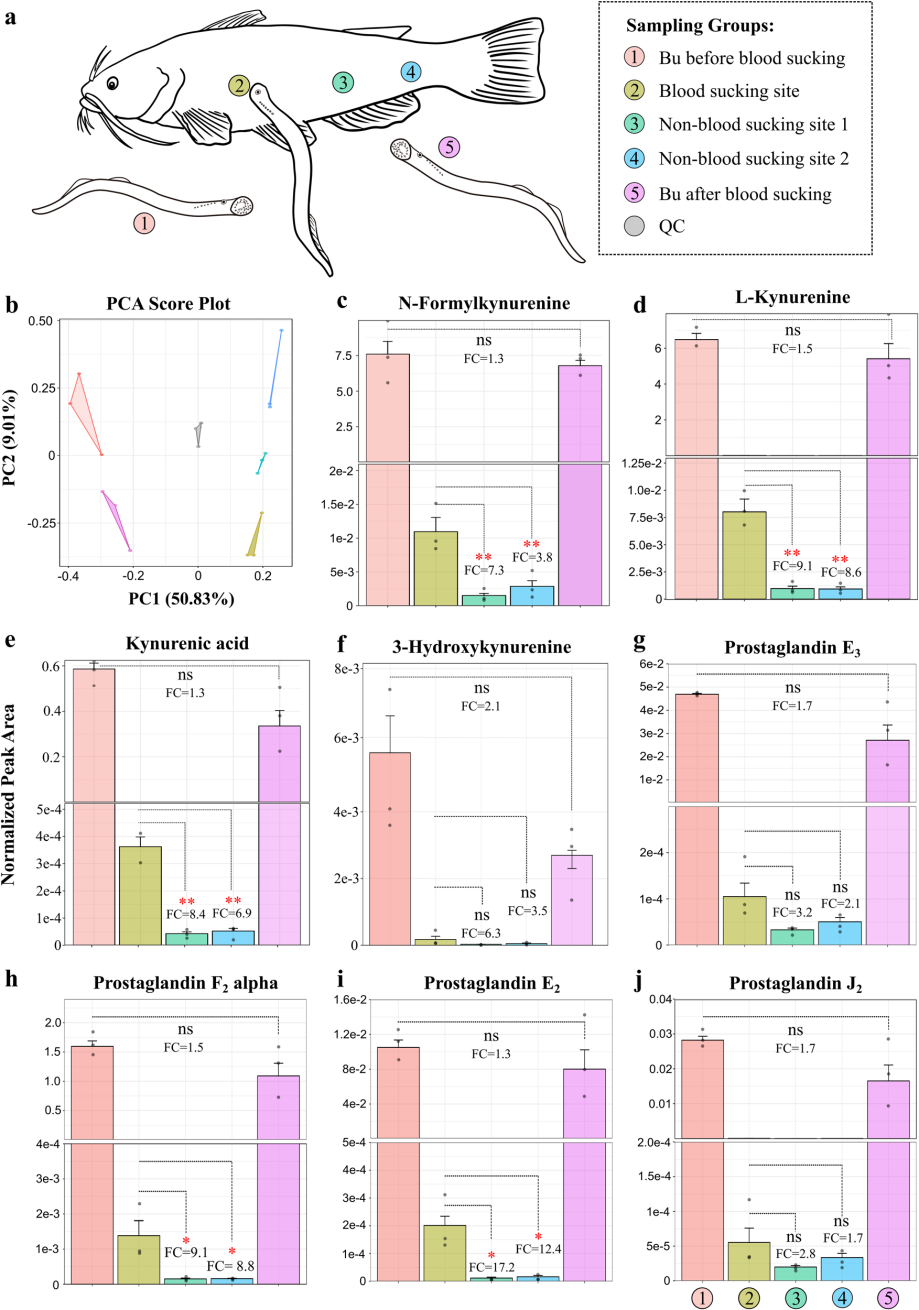
Blooding sucking experiment. **a** Five groups of lampreys were used for blood-sucking experiment. Group 1 is the buccal gland collected from lampreys before blood-sucking; Group 2 is the feeding-site from the host fish (catfish); Groups 3 and 4 are non-feeding sites from the host fish; and group 5 is the buccal glands collected from lampreys after blood-sucking. **b** Principal component analysis (PCA) score plots of the metabolic profiles of the 5 sample groups (*n* = 3 for each group). Measurement of relative peak areas of N-formylkynurenine (**c**), L-Kynurenine (**d**), kynurenic acid (**e**), 3-hydroxykynurenine (**f**), prostaglandin E_3_ (**g**), prostaglandin F_2_ alpha (**h**), prostaglandin E_2_ (**i**) and prostaglandin J_2_ (**j**). The data are shown as the mean ± SD (*n* = 3). The metabolite abundances between different groups were compared using one-way analysis of variance (ANOVA); If deemed significant (*p*-value < 0.05), post hoc multiple comparison analysis was performed with false discovery rate correction; ns, not significant; *p < 0.05; **p < 0.01.

In this study, we have identified two groups of metabolites, i.e., KP metabolites and prostaglandins, in the lamprey buccal gland that may play significant roles in lamprey blood-feeding (Supplementary Table 5). To verify that these metabolites indeed participated in lamprey blood feeding, we must make sure that they can be secreted from the lamprey buccal gland and injected into the feeding site of host fish. As such, we designed another set of experiments and performed a “targeted analysis” to monitor changes in the two groups of metabolites during lamprey blooding feeding. It is important to note that the Arctic lamprey (*Lethenteron camtschaticum*) do not feed in the adult stage. Instead juvenile Korean lamprey (*Eudontomyzon morii*) were used to attack host fishes and suck blood^13^. In brief, sixty lamprey were randomly divided into six groups (10 in each group). The buccal glands of three lamprey groups were dissected, and their secretion was collected before blood-sucking. Another three groups of lamprey were fed with catfish (*Silurus asotus*) for 20 min, and then their buccal gland secretion was collected. In addition, three sampling sites from the catfish, i.e., the blood-sucking site and two non-blood-sucking sites of the host, were collected. In total, the LCMS analyses include 5 sample groups: group 1 is the buccal gland from lamprey before feeding (BGb); group 2 is the blood-sucking site from catfish (BSS); groups 3 and 4 are control sites (non-blood-sucking sites, C1 and C2) from catfish, and group 5 is the buccal gland from lamprey after feeding (BGa) (Fig. 4a). By comparing between BGb and BGa, we could know that if the amounts of these metabolites are reduced in buccal gland after blood-sucking; and by comparing among BSS, C1, and C2, we could check if these metabolites are transferred from lamprey buccal gland to the lesion site of the host fish.

The PCA score plot of the LCMS data clearly demonstrated distinct separation among the five sample groups, indicating pronounced differences in their metabolic profiles. (Fig. 4b). The results for KP metabolites, i.e., N-formylkynurenine, L-kynurenine, kynurenic acid, and 3-hydroxykynurenine, are displayed in Fig. 4c–f. Although no significant statistical differences of the four KP metabolites were observed between BGb and BGa (FDR-adjusted *p*-value > 0.05), fold change analysis showed that the amounts of all the four metabolites were reduced after blood feeding (Fig. 4c–f), suggesting that these metabolites were released from lamprey buccal gland during blood sucking. By contrast, significant statistical differences of three KP metabolites, i.e., N-formylkynurenine, L-kynurenine, and kynurenic acid, were found between BSS and C1, and between BSS and C2 (FDR-adjusted *p*-value < 0.05) (Fig. 4c–e). Fold change analysis showed that all the four metabolites were highly accumulated in BSS compared to non-blood-sucking sites of the host fish (C1 and C2), demonstrating that the four KP metabolites were transferred from lamprey buccal gland to the sucking site of the host fish. Similarly, the results for another four KP metabolites, i.e., 3-hydroxykynurenine-O-sulfate, anthranilic acid, xanthurenic acid, and 3-hydroxyanthranilic acid, also confirmed that they could be secreted from the buccal gland and injected into the site of attachment of catfish (Supplementary Fig. S11). Although no significant statistical differences were found between the levels of PGs in BGb and BGa (FDR-adjusted *p*-value > 0.05), the amounts of all four PGs were observed to be reduced in the buccal gland following blood-sucking (Fig. 4g–j). The results also showed that all the four PGs increased in BSS compared to C1 and C2. In particular, PGF_2_ alpha and PGE2 were statistically higher in the BSS compared to C1 and C2 (Fig. 4h, i).

### Lamprey spatial metabolomics database

**Fig. 5:**
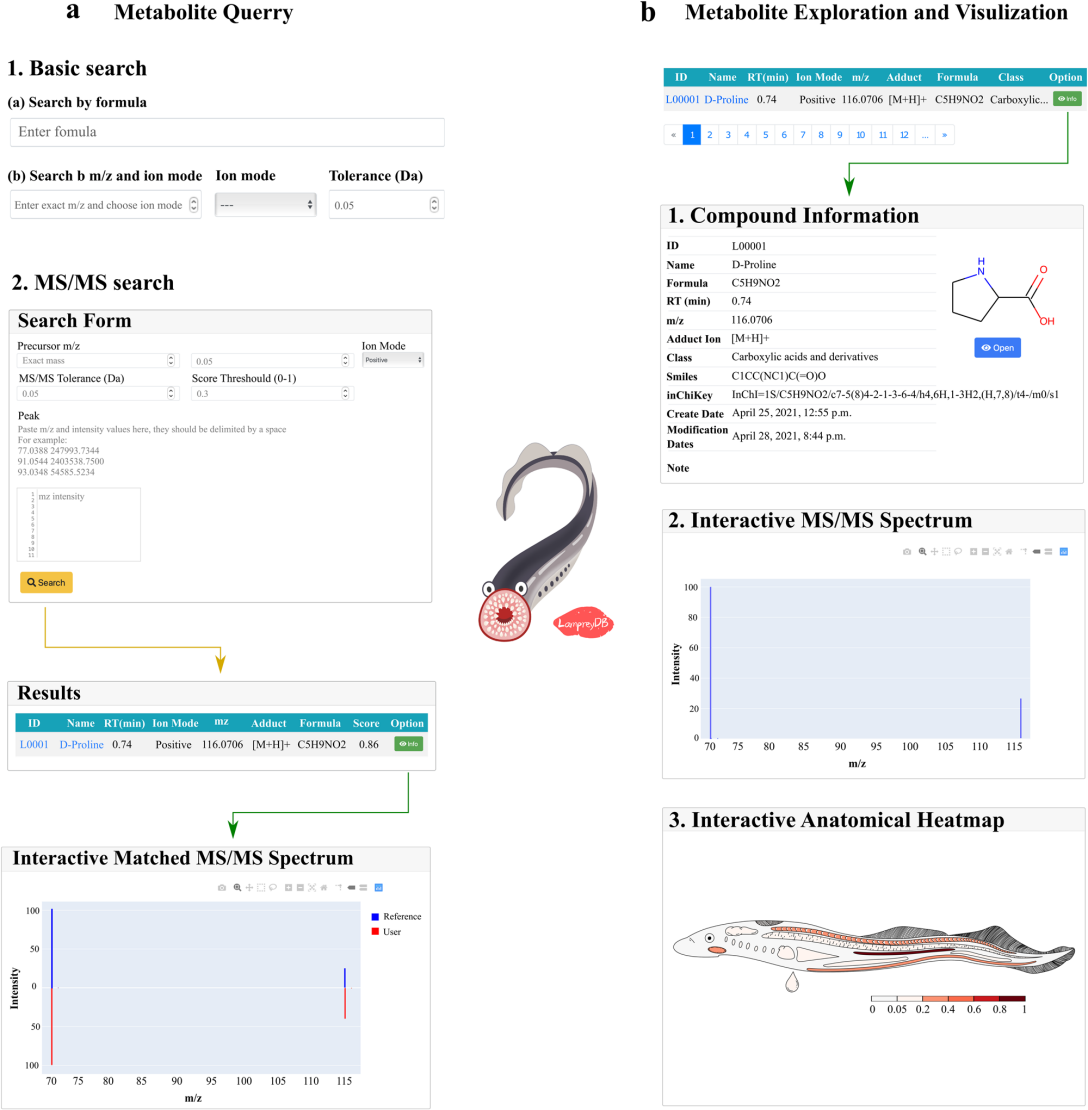
Overview of the LampreyDB spatial metabolomics database. **a** The LampreyDB allows text-based search such as using chemical formula, *m/z* value, and a list of MS/MS fragments. **b** The LampreyDB provides rich information including metabolite name, class, chemical formula, retention time, accurate *m/z* value, SMILES, inChiKey, chemical structure, interactive MS/MS spectrum and interactive anatomical heatmap for each metabolite in the database.

Due to its unique status in vertebrate evolution, lampreys have become an important animal model in diverse research fields^2,30^, such as vertebrate evolutionary and development^8,31,32^, fundamental aspects of vertebrate neurobiology^31,33^, adaptive immunity^34,35^, blood clotting^36^, and bioactive compound identification^23^. Metabolomics, as a relatively new member in the “omics” field, provides an additional powerful tool for lamprey studies. However, the lamprey-specific metabolomics database is still missing. As such, we have established LampreyDB (https://www.lampreydb.com), a tissue-wide spatial lamprey metabolomics database that contains all the identified and annotated metabolites from our experiment. LampreyDB allows users to explore lamprey-specific metabolites with text-based searches, i.e., chemical formula, *m/z* value, or a list of MS/MS fragments (Fig. 5a). The resulting summary page displays a table containing all matched metabolites from the database. Each metabolite entry is hyperlinked to an individual metabolite description page that contains the following information (if available): metabolite name, class, chemical formula, retention time, accurate *m/z* value, SMILES, inChiKey and chemical structure (Fig. 5b). Interactive MS2 spectrum plot and interactive anatomical heatmap are provided in the same page for each metabolite so that the user can easily explore the fragment peaks of each metabolite, and visually inspect and compare its spatial distribution. Currently, LampreyDB contains information on over 1000 metabolites (2031 records from both positive and negative ion modes) detected in lamprey.

## Discussion

When attempting to feed on their hosts, lampreys are challenged by the host defenses, such as hemostasis, inflammation, and immunity. Accordingly, to ensure successful and continuous blood-feeding, lampreys have evolved a complex and sophisticated cocktail of buccal gland secretion components, consisting of a variety of bioactive proteins, peptides, and metabolites, to counteract the host responses^16,23^. Recent advances in genomics, transcriptomics, and proteomics have allowed the discovery of diverse proteins exhibiting anticoagulant and vasodilator activities from the lamprey buccal gland^7,13^. However, studies on metabolites present in the lamprey buccal gland are still missing.

In this study, we applied a spatial metabolomics approach to search for lamprey blood-feeding-related metabolites. Spatial metabolomics is a field of omics research focused on detecting and interpreting metabolites in the spatial context of cells, tissues, organs, and organisms^37^. Mass spectrometry imaging (MSI) is one of the most noteworthy techniques in spatial metabolomics studies, with the achievable spatial resolution down to cellular and sub-cellular levels to date^38,39^. However, the molecular coverage is typically low and metabolite identification is challenging in MSI^40–42^. By contrast, although limited in spatial resolution, LCMS-based spatial metabolomics approach provides unprecedented sensitivity and molecular coverage, thus allowing a more detailed investigation of the biological system. As our objective is not to map the spatial distribution of metabolite in lampreys, we decided to use LCMS to perform a tissue-wide spatial metabolomics analysis. This approach allows us to explore tissue-specific metabolites in an “untargeted manner”. For instance, by comparing the metabolic profiles among different lamprey tissues, we were able to pinpoint lamprey buccal gland-specific metabolites. These metabolites are promising candidates for screening lamprey blooding-feeding-related metabolites. With this approach, we were able to detect over 1500 unique mass features that were highly accumulated in lamprey buccal gland (FC > = 10 and FDR-adjusted *p*-value < 0.05). Our result implies that the buccal gland contains a much broader complexity of small metabolites than previously anticipated. Further statistical analysis, literature search, and biological function analysis led to the identification of two groups of candidate metabolites, i.e., the KP metabolites and prostaglandins (PGs), that may be involved in lamprey blood-sucking.

The KP has been a subject of intense research activity in recent years with respect to the discovery of new and important roles its metabolites play, particularly in neuronal and immune function^25^. In addition to protein synthesis, tryptophan is the precursor of many physiologically important metabolites, whereas over 95% of tryptophan goes into KP^25,43^. To begin with, tryptophan is converted into N-formylkynurenine through the action of either tryptophan 2,3-dioxygenase (TDO) or indoleamine 2,3-dioxygenase (IDO1 and 2), and then into kynurenine by N-formylkynurenine formamidase (FAM). Kynurenine is metabolized mainly by hydroxylation to 3-hydroxykynurenine by kynurenine monooxygenase (KMO) followed by hydrolysis of 3-hydroxykynurenine to 3-hydroxyanthranilic acid by kynureninase (Fig. 3a). The KP is highly regulated in the immune system, where it promotes immunosuppression in response to infection or inflammation. Although IDO and TDO share similar functions as a mediator of tryptophan degradation, their biochemical properties are rather different. IDO is a monomeric enzyme less expressed in normal tissues and upregulated during inflammation to suppress immune reaction. In particular, IDO1 has a broad spectrum of activity on immune cells regulation, which controls the balance between stimulation and suppression of the immune system at sites of local inflammation that is relevant to a wide range of autoimmune and inflammatory diseases^26^. While TDO is a tetramer, and it is highly expressed in liver where it degrades excesses of dietary tryptophan and produces immunosuppressive KP metabolites^25^. Unlike reported in humans and many other animal species that KP and its first enzyme TDO exist mainly in liver^25^, our spatial metabolomics results disclosed that KP and TDO were exclusively located in lamprey buccal gland (Fig. 3b, c). The identification of KP in lamprey buccal gland may imply a unique mechanism for lamprey blood-feeding and parasitism: instead of evolving novel molecules, lampreys accumulate abundant KP metabolites in their buccal gland, and during blood-feeding these metabolites are injected into the bite site of the host fish, serving as immune suppressors to facilitate continuous blood-feeding. Unlike TDO which mainly existed in lamprey buccal gland, IDO was found mostly expressed in lamprey liver, and its expression level in buccal gland was negligible (Fig. 3c). Nevertheless, the main theory underlying the immunosuppressive function for these enzymes is associated with their canonical tryptophan catabolic properties: TDO and IDO-mediated depletion of tryptophan and the accumulation of kynurenine and other KP metabolites leads to the suppression of immune effector cells and the upregulation, activation, and/or induction of tolerogenic immune cells^44^. In addition, vasodilation experiment on catfish showed that apart from functioning as immune suppressor, kynurenine can also relax the blood vessels of the host fish, thus improving the blood flow of the host fish at the bite site (Fig. 3d). KP is a well-studied pathway, and the structures and functions of its pathway metabolites have been elucidated. In this study, we have also detected a rarely reported KP metabolite, namely 3-hydroxykynurenine O-sulfate, in lamprey buccal gland (Fig. 3a). This metabolite was first identified in lamprey by ref. ^23^, but it has been also reported in blood-sucking insects, such as *Rhodnius prolixus*^24^, suggesting that it is a blood-feeding related metabolite.

Prostaglandins (PGs) are eicosanoid components derived from arachidonic acid through three sequential enzymatic reactions. They act as ‘local hormones’ and regulate a plethora of physiological processes^45,46^. PGs are a group of well-studied blood-sucking related metabolites, and they have been discovered in many bloodsuckers, including ticks^47–50^, salmon louses^51,52^ and forest leeches^46^. Among them, PGE_2_ is the most commonly found PGs in the secretions, and it has demonstrated prolonged parasitic feeding (anticoagulant), increased blood flow to the site of attachment (vasodilation), and/or evasion of host immune responses (immunomodulator)^49^. Another important function of PGE_2_ is to mobilize Ca^2+^ and stimulate the secretion of anticoagulant proteins during blooding feeding^53^. It has been also reported that PGE_2_ could inhibit fibroblast migration to the feeding lesion, therefore inhibiting wound healing^49^. In addition to PGE_2_, PGF_2_ alpha was also commonly detected in bloodsuckers with reported functions of vasodilation, platelet aggregation inhibition, anti-inflammation, and pain alleviation^46,47^. In this study, we have detected another two PGs, i.e., PGJ_2_ and PGE_3_, in lamprey buccal gland. Both PGJ_2_ and PGE_3_ are known to have anti-inflammatory property^54^. Although they are not reported in other blood-sucking species, it is tempting to speculate a function of the two PGs in blood-feeding and parasitism. Interestingly, all the four PGs were exclusively accumulated in lamprey buccal gland (Fig. 3f), while its precursor arachidonic acid was found in most tissues (Supplementary Fig. 12). The detection of PGs in lampreys suggested that lampreys may share a similar blood-sucking strategy as other bloodsuckers.

While our focus in this study is on blood-feeding related metabolites in lampreys, it is worth noting that lampreys contain a wide range of metabolites that serve various biologically and physiologically important functions. For instance, we identified a sulfated bile acid named petromyzonol sulfate, which acts as a unique sex pheromone to lampreys^55,56^, and its distributions appear to be particularly tissue-specific (Supplementary Fig. 13). Since spatially resolved metabolic information is of great benefit to many studies using lamprey as an animal model, we have created a lamprey spatial metabolomics database (https://www.lampreydb.com), which includes detailed qualitative, quantitative, and spatial distribution information of each metabolite. Users can easily query and check their metabolites of interest, and/or identify unknown peaks from this database. The current LampreyDB version includes information on over 1000 metabolites (2031 records from both positive and negative ion modes). We are now applying a fractionation-assisted NMR-based metabolomics approach to identify the unknown mass peaks from different lamprey tissues so that we can increase the number of metabolites and enhance the quality and reliability of the information in LampreyDB in the near future.

From a bloodsucker’s perspective, paradise is a place where the host blood does not clot, the blood flow is intense at the feeding site, and the host will not resist or harm the guests. While reality is different. The vertebrate hosts are equipped with three efficient weapons that fight against blood-feeding behaviors: hemostasis, inflammation, and immunity^57^. Blood-sucking animals have evolved many different strategies to succeed against all the complex barriers imposed by their hosts. Studies have shown that almost all blood-sucking arthropods have at least one anticlotting, one vasodilator, and one antiplatelet component, and in many cases, more than one substance is present in each category. In our study, we showed that in addition to relying on several active proteins and peptides, lampreys also secret many metabolites from their buccal glands to counteract the host responses and ensure successful and continuous blood-feeding. It has long been known that most KP metabolites are immune suppressors, and our study showed that kynurenine also functions as a vasodilator in lampreys. Although KP has been well-studied, one striking difference in our study is that the KP metabolites and the first enzyme TDO are found exclusively present in the buccal gland, which is different from reported in other animals and human that KP metabolites and TDO mostly exist in liver. This may imply a unique blood-feeding mechanism in lampreys. PGs are a group of well-known blood-sucking-related metabolites in other bloodsucking animals. They have been demonstrated to possess multiple roles to assist blood-feeding, such as increasing vasodilation, suppressing inflammation, and inhibiting wound healing. The identification of PGs in lampreys suggests that all bloodsuckers may share some similar blood-feeding strategies. These findings demonstrate the complex nature of lamprey buccal gland and highlight the diversity in the mechanisms utilized for blood-sucking in lampreys.

## Methods

### Chemicals

All chemicals and solvents were of analytical or HPLC grade. Water, methanol, acetonitrile, formic acid were purchased from CNW Technologies GmbH (Düsseldorf, Germany). L-2-chlorophenylalanine was from Shanghai Hengchuang Bio-technology Co., Ltd. (Shanghai, China). L-tryptophan was purchased from Sangon (Shanghai, China), and L-kynurenine from Macklin (Shanghai, China).

### Lamprey model and ethical approval

The adult Arctic lamprey (*Lethenteron camtschaticum*) at spawning migration stage were obtained in December 2020 in Songhua River in Heilongjiang province of China. Fourteen different lamprey tissues, i.e., heart, liver, kidney, brain, supraneural body, muscle, intestine, gill, eye, testis, ovary, buccal gland, blood, and notochord, were carefully dissected and washed in sterile phosphate buffered saline (PBS: 10 mM phosphate buffer, 2.7 mM potassium chloride, 137 mM sodium chloride, pH 7.4). The secretion of lamprey buccal gland was collected through a syringe. All the samples were snap frozen in liquid N_2_ and stored at −80 °C before LCMS analysis.

The parasitic juvenile Korean lamprey (*Eudontomyzon morii*) were obtained in December 2020 in Yalu River in Liaoning province of China, and they were used for blood-feeding experiment. Sixty lamprey were randomly divided into six groups (10 in each group) and kept in fresh water at 10 ± 2 °C in dim light without feeding for 72 h. The buccal glands of three lamprey groups were dissected, and their secretion was collected through syringes before blood-sucking. Another three groups of lamprey were fed with catfish (*Silurus asotus*) for 20 min, and then their buccal gland secretion was collected. In addition, three sampling sites from the catfish, i.e., the blood-sucking site and two none-blood-sucking sites of the host, were collected. In total, the LCMS analyses include 5 sample groups: group 1 is the buccal gland from lamprey before feeding (BGb); group 2 is the blood-sucking site from catfish (BSS); group 3 and 4 are control sites (non-blood-sucking sites, C1 and C2) from catfish, and group 5 is the buccal gland from lamprey after feeding (BGa). All the tissues were stored at −80 °C before metabolite extraction.

The handling of lampreys and catfish was approved by the Animal Welfare and Research Ethics Committee of the Institute of Dalian Medical University (Permit number: AEE17013).

### LC-MS analysis

To extract the samples, 30 mg of each sample, 20 μL IS (L-2-chlorophenylalanine, 0.3 mg/mL) and 400 μL extraction solution (80% methanol/water, v/v) were added into a 2 mL Eppendorf tube, followed by adding two small steel balls. After precooling the tube at −20 °C for 2 min, each sample was ground at 60 Hz for 2 min using a Tissuelyser-48 grinding miller (Jingxing Limited Company, Shanghai, China). The resulting extract was briefly vortexed and sonicated at ambient temperature (25–28 °C) for 10 min. Subsequently, the extracts were centrifuged at 13,000 rpm and 4 °C for 10 min. Next, 300 μL of the supernatant was transferred into a brown glass vial and dried using a freeze concentration centrifugal dryer. To each sample, 300 μL of a methanol and water mixture (1/4, v/v) was added. The mixture was vortexed for 30 s and then placed at −20 °C for 2 h. Afterward, the samples were centrifuged at 13,000 rpm and 4 °C for 5 min. The resulting supernatants (150 μL) from each tube were collected using crystal syringes, filtered through a 0.22 μm PTFE filter (Acrodisc® CR 13 mm; PALL), and transferred to LC vials for LCMS analysis. Pooled QC samples were prepared by combining aliquots of 20 μL from each extracted sample.

A Dionex Ultimate 3000 UHPLC system fitted with Q-Exactive quadrupole-Orbitrap mass spectrometer equipped with heated electrospray ionization (ESI) source (Thermo Fisher Scientific, Waltham, MA, USA) was used for spatial metabolomics analysis in both positive and negative ion modes. An ACQUITY UPLC HSS T3 column (1.8 μm, 2.1 × 100 mm) was employed. The binary gradient elution system consisted of (A) water (containing 0.1% formic acid, v/v) and (B) acetonitrile (containing 0.1% formic acid, v/v) and separation was achieved using the following gradient: 5% B over 0–2 min, 5–25% B over 2–4 min, 25–50 B over 4–8 min, 50–80% B over 8–10 min, 80–100% B over 10–14 min, the composition was held at 100% B for 1 min, then 15–15.1 min, 100% to 5% B, and 15.1–18 min holding at 5% B. The flow rate was 0.35 mL/min and column temperature was 45 °C. All samples were kept at 4 °C during analysis. The injection volume was 2 μL. The mass range was from *m/z* 66.7 to 1000.5. The resolution was set at 70,000 for the full MS scans and 35,000 for HCD MS/MS scans. The Collision energy was set at 10, 20 and 40 eV. The mass spectrometer operated as follows: spray voltage, 3800 V (+) and −3000 V (−); sheath gas flow rate, 35 arbitrary units; auxiliary gas flow rate, 8 arbitrary units; capillary temperature, 320 °C. The QCs were injected at regular intervals (every 10 samples) throughout the analytical run to provide a set of data from which repeatability can be assessed.

### LCMS data processing

Raw data quality was first checked using R package RawHummus^58^ on QC samples in both positive and negative ion modes. The resulting QC reports are shown in Supplementary Data 1–2. Data pre-processing and metabolite identification were performed using three different software tools, i.e., Compound Discoverer (v.3.3, Thermo Scientific), Progenesis QI (v.2.3, Waters), and MS-DIAL (v.4.0)^19,20^. Of which, Progenesis QI and MS-DIAL were mainly used for metabolite identification purpose. For each data processing software, multiple parameters were tuned, and the optimized settings were summarized in Supplementary Table 1–3.

### Lamprey spatial metabolomics database construction

The LampreyDB database (https://www.lampreydb.com) was organized with MySQL (v.8.0) and Django (v.3.0.6). The web interface was developed using HTML with JavaScript. The interactive anatomy heatmap was produced with home written script and Python package beautiful soup (https://pypi.org/project/beautifulsoup4/). Other figures such as MS spectrum plots were produced using Python package plotly (https://plotly.com/python/). LampreyDB is hosted on Microsoft Azure cloud service.

### Gene cloning and bioinformatics analysis

The full-length open reading frames (ORF) of the *Lj-TDO* (tryptophan 2,3-dioxygenase), *Lj-AADAT* (aminoadipate aminotransferase), *Lj-IDO* (indoleamine 2,3-dioxygenase) and *Lj-KMO* (kynurenine monooxygenase) genes were obtained by PCR. Primer Premier 5.0 was used to design specific PCR primers in open reading frame. The sequences of all primers used for gene syntheses were listed in Supplementary Table 4. The amino acid sequences of TDO, AADAT, IDO), and KMO were obtained from the National Center for Biotechnology Information (NCBI) database (https://www.ncbi.nlm.nih.gov/) and stored in the FASTA format. These sequences were utilized for sequence alignments and subsequent bioinformatics analyses. Sequence alignment was carried out using ClustalW software. The resulting aligned sequences were utilized to construct phylogenetic trees via the Neighbor-Joining (NJ) method, with 1000 bootstrapped replicates implemented in MEGA-X software^59^. Pairwise deletion option was utilized during the analysis to account for gaps and missing data. The conserved motifs were procured from online multiple expectation maximization (MEME, http://meme-suite.org/tools/meme) and the number of motifs was select: 15. The output search results were drawn with TBtools software. Conserved functional protein domains were predicted with Simple Modular Architecture Research Tool (SMART, http://smart.embl-heidelberg.de/). To better understand the evolution of gene family between jawless and jawed vertebrates, neighboring genes environment of TDO, AADAT, IDO, and KMO of representative animals were conducted by using the Genomicus online tool (https://www.genomicus.bio.ens.psl.eu/genomicus-92.01/cgi-bin/search.pl) and the Ensembl database (https://www.ensembl.org/index.html). The gene structure analysis was performed by using the Ensembl database. SWISS-MODEL (http://swissmodel.expasy.org/interactive) online was used to predict the 3D structure of Lj-TDO, Lj-AADAT, Lj-IDO and Lj-KMO.

### Real-time quantitative PCR analysis

The total RNA samples were isolated separately from the leukocytes, ovary, brain, livers, muscles, gills, oral glands, intestines, hearts, kidney, testis and supraneural bodies by using RNAiso reagent (TaKaRa, China). Leukocytes were collected from circulated blood. The cDNA was then synthesized using HiScript®II Q Select RT SuperMix for qPCR (+gDNA wiper) Kit (Vazyme, China) according to the manufacturer’s protocol. The specific primers for qPCR were designed using Primer Premier 5.0 program and the primer sequences were shown in Supplementary Table 4. The qPCR was carried out in 20 µL reactions of 2 × chamQ SYBR Color qPCR Master Mix (low ROX Rremixed) (Vazyme Biotech, China), forward primer (10 µM), reverse primer (10 µM) and cDNA 200 ng. Quantitative real-time PCR was performed with qTOWER 2.0 Real Time PCR System and the following program: 30 s at 95 °C, 40 cycles of 10 s at 95 °C and 30 s at 60 °C. The change in threshold cycle (∆CT) was calculated by subtracting the average CT of *Lj-GAPDH* mRNA from the average CT of the target genes. All experiments were performed in triplicate.

### Vasomotor reactivity assessment

The vasomotor reactivity was measured by adapting the protocol described elsewhere^27^. In brief, catfish aortas were dissected, cut into 1.8–2.0 mm aortic ring, and placed in a culture dish. The viability was then confirmed by incremental constriction to high potassium solutions (KCl) with the final concentration of 60 mmol/L. Aortic rings were next pre-incubated with 60 mmol/L KCl, pre-constricted to the maximum response, and then the blood vessel diameters were measured under a stereoscopic microscope. Finally, the vessel diameters were recorded 15 min after addition of different concentrations of kynurenine solutions (i.e., 2 mM, 4 mM, and 8 mM). PBS was added as the blank control group, and all the measurements were repeated five times (*n* = 5).

### Statistics and reproducibility

All processing and null hypothesis testing were performed using R^60^ (version 4.2.3). To compare the relative metabolite abundance among different tissue groups, one-way analysis of variance (ANOVA) was performed; If deemed significant (*p*-value < 0.05), post hoc multiple comparison analysis was performed with false discovery rate correction. PCA was performed with R package MSBox (https://cran.r-project.org/web/packages/MSbox/index.html); heatmap and hierarchical clustering analysis were performed using R package pheatmap (https://cran.r-project.org/web/packages/pheatmap/index.html). Other figures such as bar plots and line plots were produced using R package ggplot2^61^ and ggbreak^62^. Data are represented as means ± SD of the mean (*n* = 3).

### Reporting summary

Further information on research design is available in the Nature Portfolio Reporting Summary linked to this article.

### Supplementary information

Supplementary Information

Description of Additional Supplementary Files

Supplementary Data 1

Supplementary Data 2

Supplementary Data 3

Supplementary Data 4

Reporting summary

## Data Availability

The sequences files used in this study are available in the National Center for Biotechnology Information (accession number: ON814546, ON814547, ON814548, and ON814549). The metabolomics data have been archived in MetaboLights with the identifier of MTBLS5857. The spatial metabolomics database for lampreys is publicly accessible at https://www.lampreydb.com. Numerical source data underlying all graphs and charts can be found in Supplementary Data 3 and 4.
