# Supplementary material for: Spatial Metabolomics Reveals the Multifaceted Nature of Lamprey Buccal Gland and Its Diverse Mechanisms for Blood-Feeding
Source: Commun Biol. 2023 Aug 28;6:881. doi: 10.1038/s42003-023-05250-x (PMC10462737; doi:10.1038/s42003-023-05250-x)

Spatial Metabolomics Reveals the Multifaceted Nature of Lamprey Buccal Gland and Its Diverse Mechanisms for Blood-Feeding

Meng Gou[1,2#], Xuyuan Duan[1,2#], Jun Li[1,2#] , Yaocen Wang[1,2], Qingwei Li[1,2], Yue Pang[1,2*], Yonghui Dong[3*]

[1]College of Life Science, Liaoning Normal University, Dalian 116081, China

[2]Lamprey Research Center, Liaoning Normal University, Dalian 116081, China

[3]Weizmann Institute of Science, Rehovot, Israel

[#] These authors contributed equally to this work

[*]To whom correspondence may be addressed.

E-mail: pangyue@lnnu.edu.cn; yonghui.dong@weizmann.ac.il

Table of Content

1. Table S1. The optimal set of parameters for Compound Discoverer

2. Table S2. The optimal set of parameters for Progenesis QI

3. Table S3. The optimal set of parameters for MS-DIAL

4. Table S4. The primer sequences used in the study

5. Table S5. Putative kynurenine pathway metabolites and prostaglandins identified in the buccal gland of lamprey.

6. Fig. S1 Head-to-tail plot of experimental and library ESI-MS/MS spectra of tryptophan.

7. Fig. S2 Head-to-tail plot of experimental and library ESI-MS/MS spectra of L-kynurenine.

8. Fig. S3 Head-to-tail plot of experimental and library ESI-MS/MS spectra of L-kynurenic acid.

9. Fig. S4 Head-to-tail plot of experimental and library ESI-MS/MS spectra of 3-hydroxyanthranilic acid.

10. Fig. S5 Head-to-tail plot of experimental and library ESI-MS/MS spectra of xanthurenic acid.

11. Fig. S6 Head-to-tail plot of experimental and library ESI-MS/MS spectra of anthranillic acid.

12. Fig. S7 Head-to-tail plot of experimental and library ESI-MS/MS spectra of prostaglandin E2.

13. Fig. S8 Head-to-tail plot of experimental and library ESI-MS/MS spectra of prostaglandin E3.

14. Fig. S9 Head-to-tail plot of experimental and library ESI-MS/MS spectra of prostaglandin F2α.

15. Fig. S10 Head-to-tail plot of experimental and library ESI-MS/MS spectra of prostaglandin J2.

Table S1. The optimal set of parameters for Compound Discoverer

Parameter	Value
Alignment/ RT tolerance (min)	2
Select spectra/ min peak count	1
Select spectra/ S/N threshold (FT-only)	1.5
Detect compound/ Intensity	1000,000
Peak detection/ max peak width (min)	0.5
Peak detection/ min scan per peak	3
Gap filling/ S/N threshold	3
Gap filling/ RT tolerance (min)	0.1

Table S2. The optimal set of parameters for Progenesis QI

Parameter	Value
Alignment/ RT tolerance (min)	2
Select spectra/ min peak count	1
Select spectra/ S/N threshold (FT-only)	1.5
Detect compound/ Intensity	1000,000
Peak detection/ max peak width (min)	0.5
Peak detection/ min scan per peak	3
Gap filling/ S/N threshold	3
Gap filling/ RT tolerance (min)	0.1

Table S3. The optimal set of parameters for MS-DIAL

Parameter	Value
Minimum peak width (scan)	5
Minimum peak height (amplitude)	100,000
Mass slice width (Da)	0.1
Sigma window value	0.5

Table S4. The primer sequences used in the study

Gene name	Primer sequence (5’-3’)
IDO F1	CAAGGATTACAACATACACG
IDO R1	TCCACCCAGATAGGAAGAT
IDO F2	GGGCATCATTGGCTTGG
IDO R2	GAGATAGCCCTCTTTGAACT
KMO F1	CAGGATTTGCGAGCGGTCAT
KMO R1	GAAGGAGTGGTCGTTGTTGG
KMO F2	CACATTCCGACGCTCCA
KMO R2	TTGCGGAATAGGAACCAG
KMO F3	ACTGCTTGGTGTTTGATGAACTAATGG
KMO R3	GGATGAGCCTCCTGGTATGT
AADAT F1	TCACACATCTTCAATCACAGGCGGG
AADAT R1	GGGTTACCACCATTGGGGACTGT
AADAT F2	TGGACACGCCAACATACC
AADAT R2	TCTGTGAGAAGGTGGACGT
AADAT F3	ATCCGCACAGATTCCTTC
AADAT R3	GTGGGAAATTCTGGTTCTTGTTG
TDO F1	GGACTTGGACGATGGTTTG
TDO R1	GCAAGCCTCTTCCTCCAAC
TDO F2	GCGTGCCGTACAACAAG
TDO R2	GTTGAAGAGGTCCACGAA
TDO F3	TCAACAGCCTTATGGATCTCG
TDO R3	TGAACAGACCTACTACTCCAACA
qIDO F	CGAATGCGGGAGTACATGCTACC
qIDO R	GCGAAACAAAGTGGCGAATGGAG
qKMO F	CGACTTCTTCAGCCGATACTTCCC
qKMO R	AGAGCACTTGACCGAGACCAGAG
qAADAT F	CATCCCTGAGGTGATTGACTGGTTC
qAADAT R	ACGGTCATCTCGGTGTCTGTGG

qTDO F	GTGGACTTGGACGATGGTTTGGAG
qTDO R	TGGTGTTGTGATGCTTGGTGCTC

Table S5. Putative kynurenine pathway metabolites and prostaglandins identified in the buccal gland of lamprey.

Name	Elemental composition	Retention Time (min)	Theoretical m/z	Measured m/z	ppm	Identification level
L-Tryptophan	$C_{11}H_{12}N_2O_2$	4.09	205.0971	205.0982	5.1	1
N-formylkynurenine	$C_{11}H_{12}N_2O_4$	2.22	237.0870	237.0867	-1.2	2
L-Kynurenine	$C_{10}H_{12}N_2O_3$	5.57	209.0921	209.0921	0	1
Kynurenic acid	$C_{10}H_7NO_3$	4.48	190.0499	190.0497	-0.9	2
3-Hydroxykynurenine	$C_{10}H_{12}N_2O_4$	2.60	225.0870	225.0869	-0.37	2
Anthranilic acid	$C_7H_7NO_2$	2.93	138.055	138.055	0	2
Quinolinic acid	$C_7H_5NO_4$	0.78	166.0146	166.0144	-1.1	2
Xanthurenic acid	$C_{10}H_7NO_4$	4.23	206.0448	206.0447	-0.41	2
3-Hydroxyanthranilic acid	$C_7H_7NO_3$	3.33	154.0499	154.0498	-0.45	2
L-3-hydroxykynurenine-O-sulfate2	$C_{10}H_{12}O_7N_2S$	1.29	303.0292	303.0291	-0.49	2
Prostaglandin $F_{2\alpha}$	$C_{20}H_{34}O_5$	7.58	353.2333	353.2333	0	2
Prostaglandin J_2	$C_{20}H_{30}O_4$	9.55	333.2071	333.2057	-4.2	2
Prostaglandin E_2	$C_{20}H_{32}O_5$	7.66	351.2177	351.2162	-4.27	2
Prostaglandin E_3	$C_{20}H_{30}O_5$	7.60	349.2020	349.2020	0	2

Note:

1. Metabolite identification confidence level for each identified metabolite in this study was assigned following the modified four-level classification scheme from the Metabolomics Standards Initiative[1]: **Level 1.** Validated Identification based on reference standard, with MS, MS/MS and retention time matching. **Level 2.** Putative identification based on matching fragmentation data to metabolite MS/MS libraries. **Level 3**. Tentative structures match precursor *m/z* to a metabolite database. **Level 4.** Unique molecular formula determined based on isotope abundance distribution, charge state and adduct ion determination.
2. The MS/MS spectrum of the metabolites are shown in Figure S1 and S10.
3. The Elemental composition, retention time, theoretical *m/z*, measured *m/z*, fragments, and chemical structure of other metabolites can be found in https://www.lampreydb.com.

Fig. S1 Head-to-tail plot of experimental and library ESI-MS/MS spectra of tryptophan.

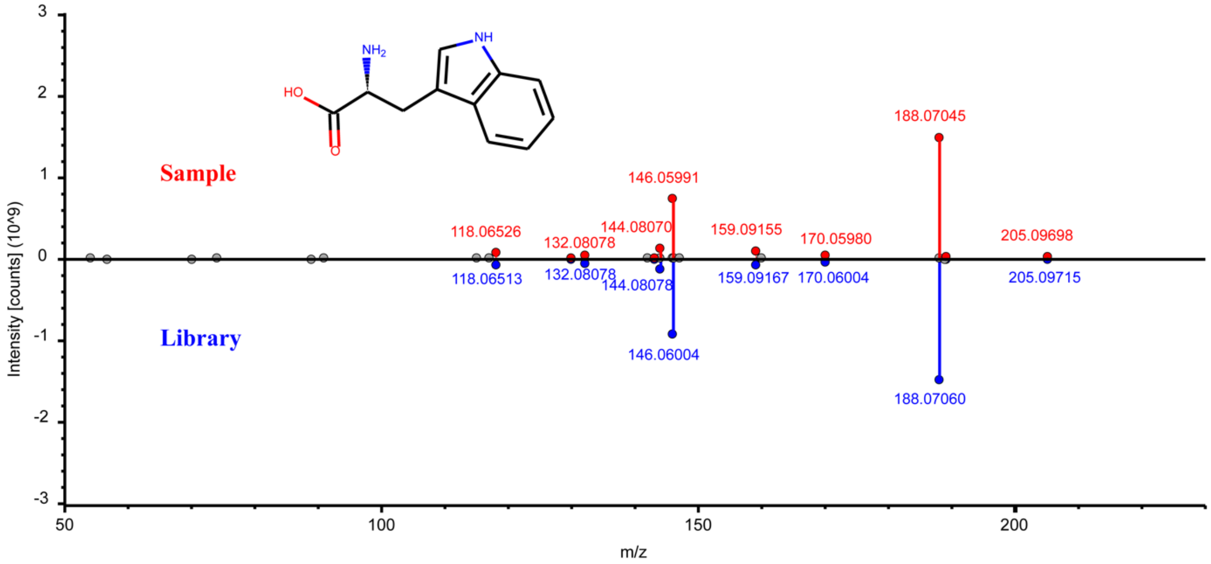

Fig. S2 Head-to-tail plot of experimental and library ESI-MS/MS spectra of L-kynurenine.

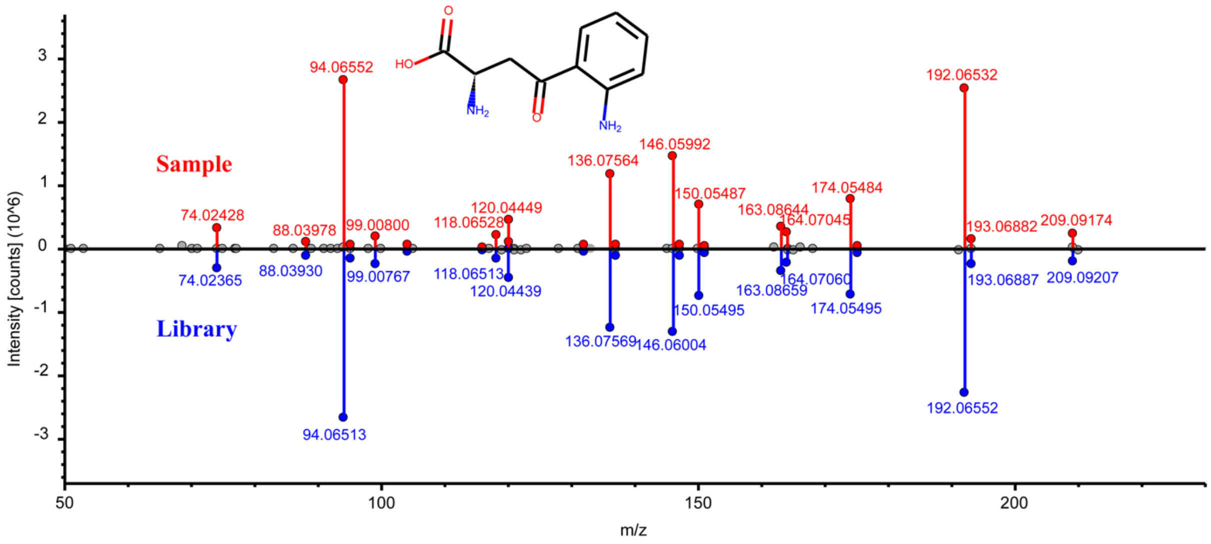

Fig. S3 Head-to-tail plot of experimental and library ESI-MS/MS spectra of L-kynurenic acid.

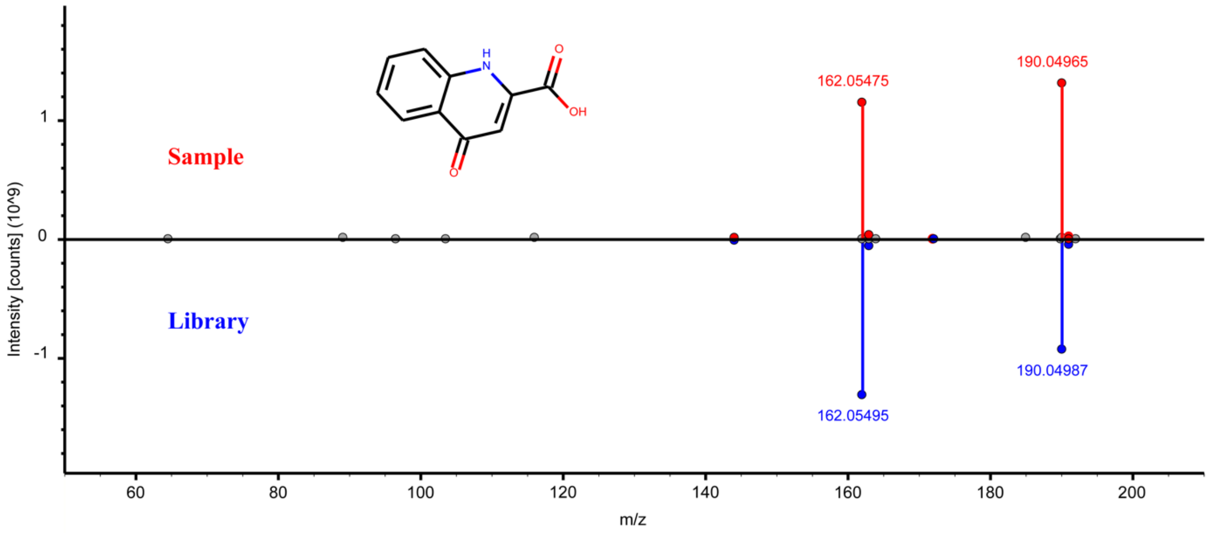

Fig. S4 Head-to-tail plot of experimental and library ESI-MS/MS spectra of 3-hydroxyanthranilic acid.

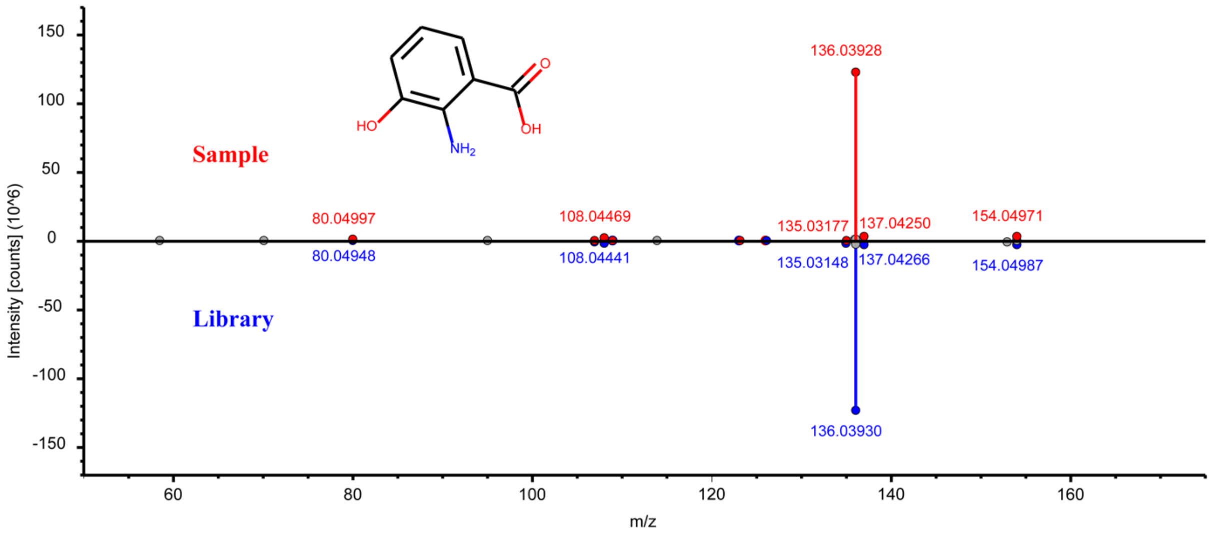

Fig. S5 Head-to-tail plot of experimental and library ESI-MS/MS spectra of xanthurenic acid.

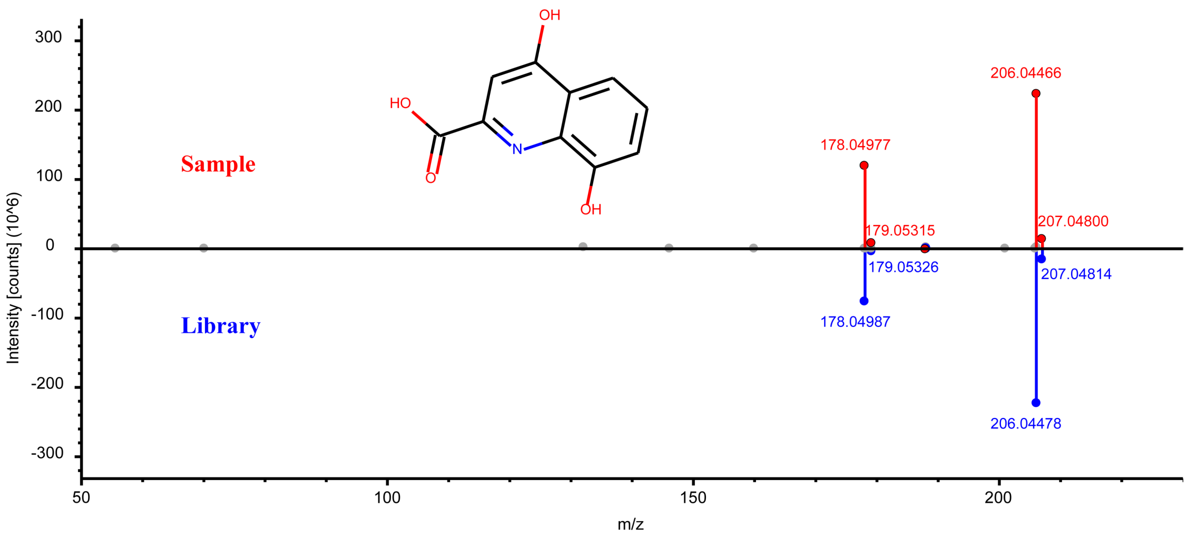

Fig. S6 Head-to-tail plot of experimental and library ESI-MS/MS spectra of anthranillic acid.

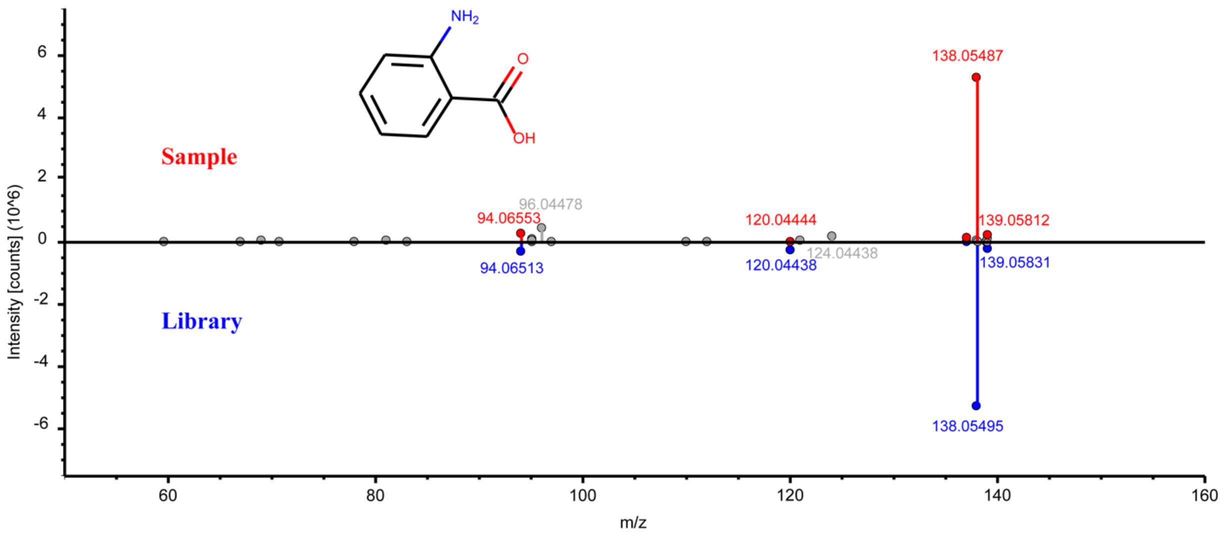

Fig. S7 Head-to-tail plot of experimental and library ESI-MS/MS spectra of prostaglandin E2.

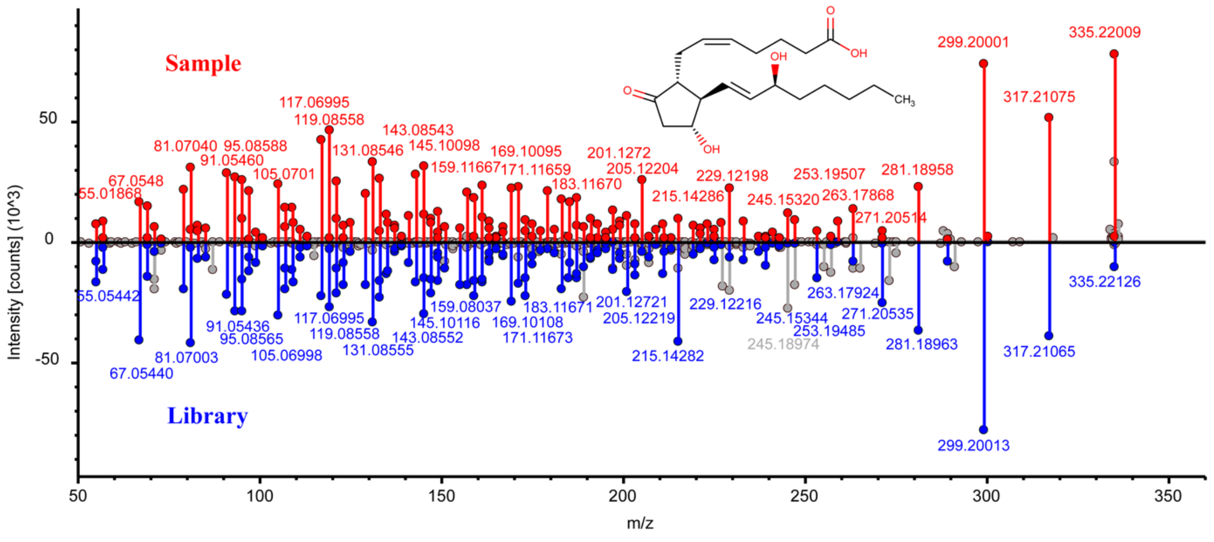

Fig. S8 Head-to-tail plot of experimental and library ESI-MS/MS spectra of prostaglandin E3.

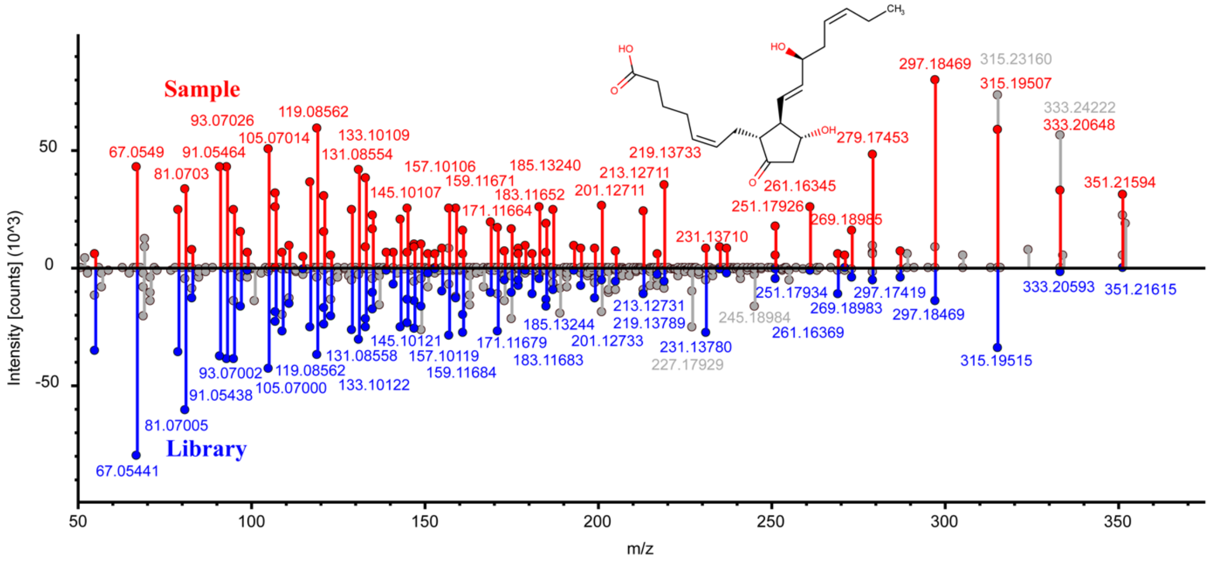

Fig. S9 Head-to-tail plot of experimental and library ESI-MS/MS spectra of prostaglandin F2α.

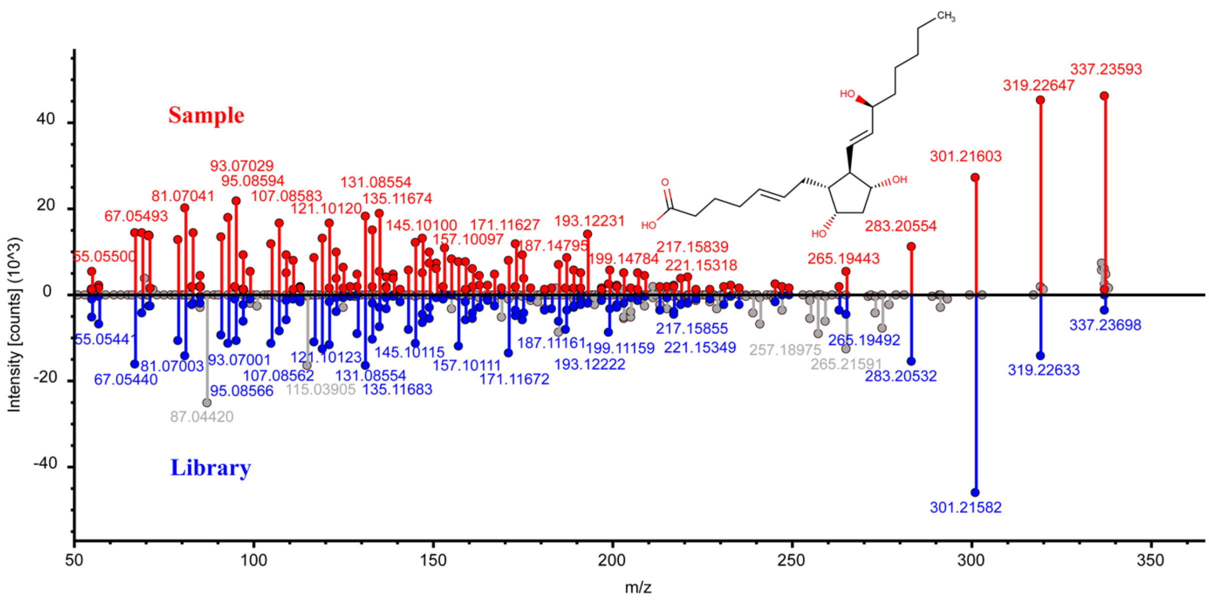

Fig. S10 Head-to-tail plot of experimental and library ESI-MS/MS spectra of prostaglandin J2.

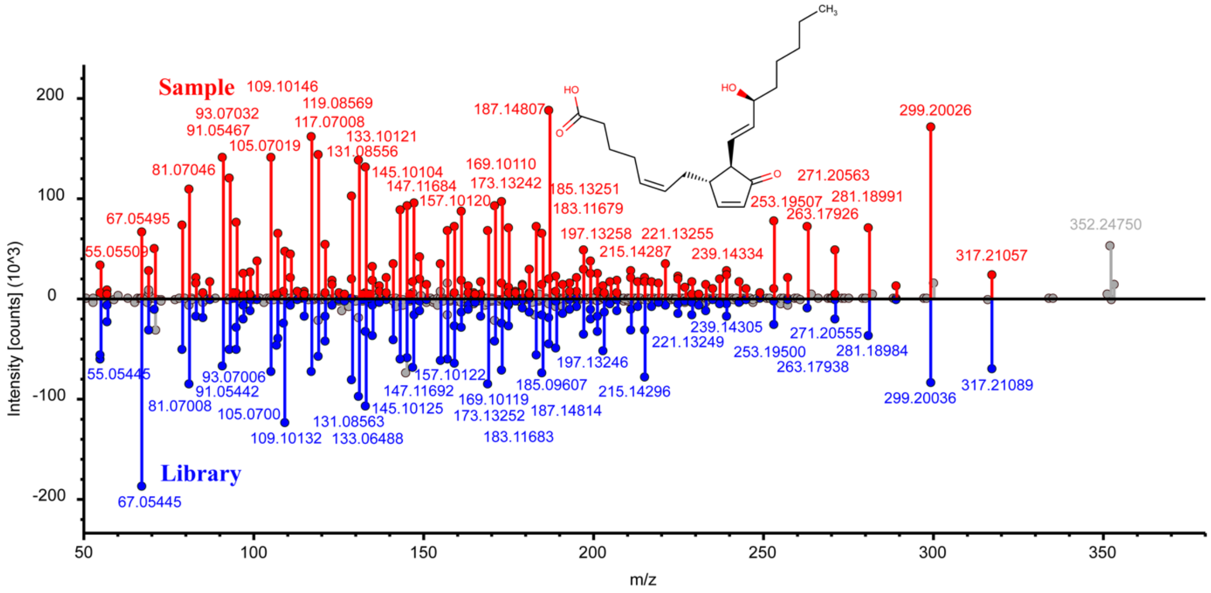

Fig. S11 Experimental design for lamprey blooding sucking. **a**, Five groups of lampreys were used for blood-sucking experiment. Group 1 is the buccal gland collected from lampreys before blood-sucking; Group 2 is the feeding-site from the host fish (catfish); Groups 3 and 4 are non-feeding sites from the host fish; and group 5 is the buccal glands collected from lampreys after blood-sucking. **b**, Principal component analysis (PCA) score plots of the metabolic profiles of the 5 sample groups (n = 3 for each group). **c-e**, Measurement of relative peak areas of anthranilic acid (c), 3-hydroxyanthranilic acid (d), xanthurenic acid (e). The data are shown as the mean ± SD (n = 3). Asterisks denote significant differences (ns, not significant; *, $p < 0.05$; **, $p < 0.01$).

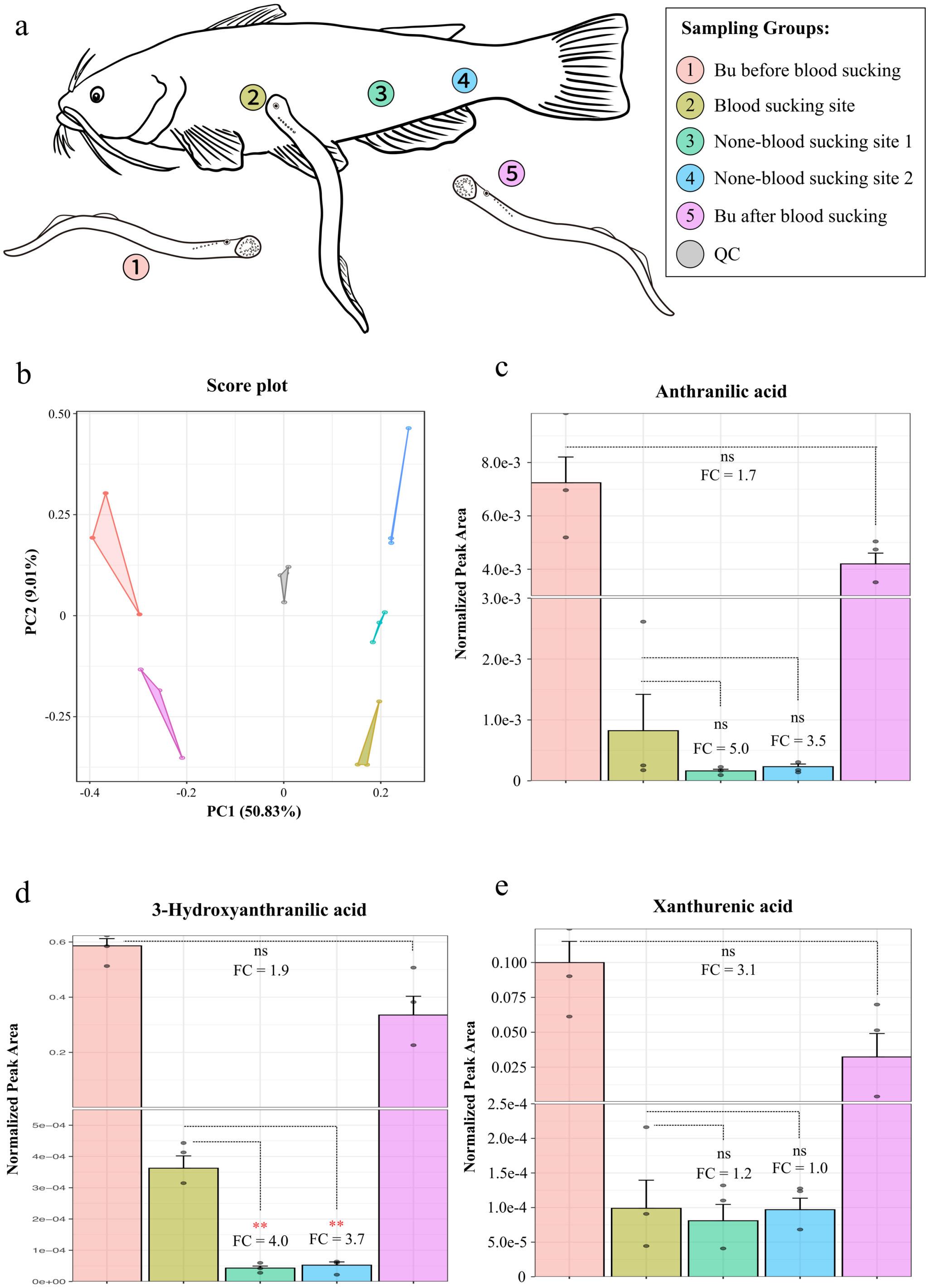
a
Sampling Groups:
1 Bu before blood sucking
2 Blood sucking site
3 None-blood sucking site 1
4 None-blood sucking site 2
5 Bu after blood sucking
QC
b
Score plot
PC2 (9.01%)
PC1 (50.83%)
c
Anthranilic acid
Normalized Peak Area
ns
FC = 1.7
FC = 5.0
FC = 3.5
d
3-Hydroxyanthranilic acid
Normalized Peak Area
ns
FC = 1.9
**
FC = 4.0
FC = 3.7
e
Xanthurenic acid
Normalized Peak Area
ns
FC = 3.1
FC = 1.2
FC = 1.0

Fig. S12 Identification of arachidonic acid in lampreys. **a,** Head-to-tail plot of experimental and library ESI-MS/MS spectra of arachidonic acid. **b**, Spatial distribution of arachidonic acid in lampreys.

a

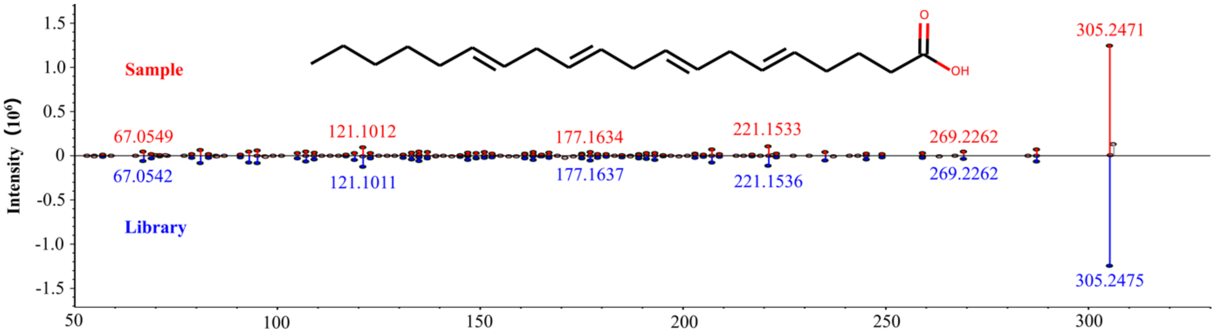

b

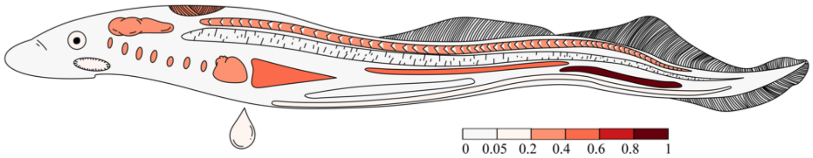

Fig. S13 Identification of petromyzonol sulfate in lampreys. **a,** Head-to-tail plot of experimental and library ESI-MS/MS spectra of petromyzonol sulfate. **b**, Spatial distribution of petromyzonol sulfate in lampreys.

a

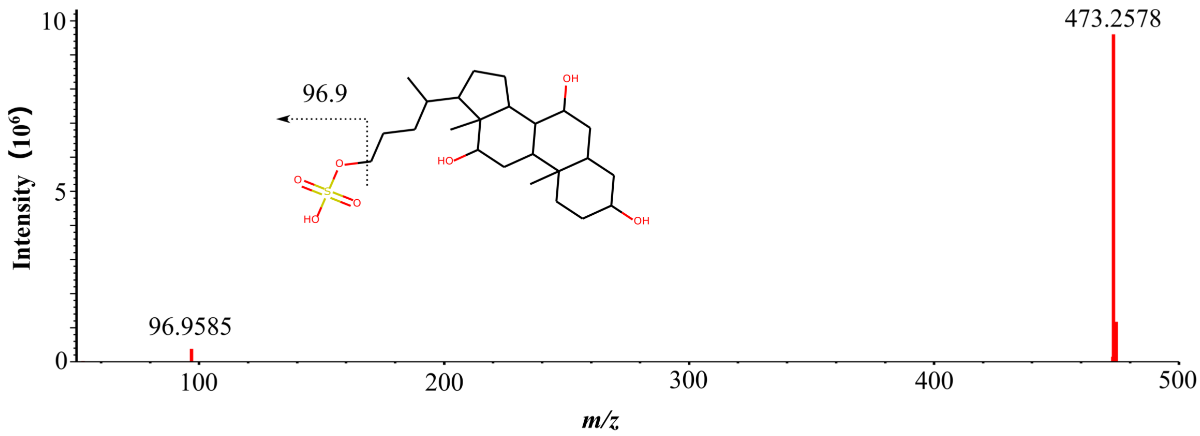

b

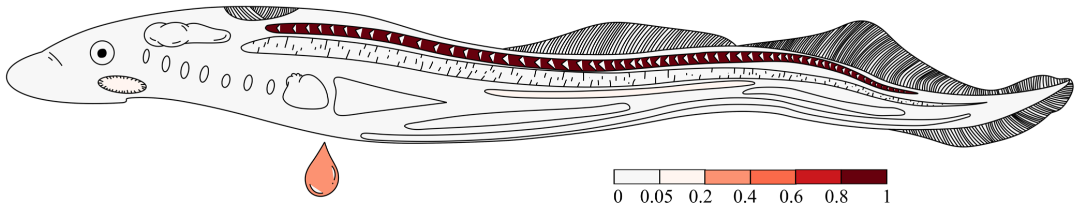

Supplement: Supplementary file 1 — Supplementary Information [file 42003_2023_5250_MOESM1_ESM.pdf]
