# Supplementary material for: Spatial Metabolomics Reveals the Multifaceted Nature of Lamprey Buccal Gland and Its Diverse Mechanisms for Blood-Feeding
Source: Commun Biol. 2023 Aug 28;6:881. doi: 10.1038/s42003-023-05250-x (PMC10462737; doi:10.1038/s42003-023-05250-x)
Supplement: Supplementary file 2 — Description of Additional Supplementary Files [file 42003_2023_5250_MOESM2_ESM.pdf]

## Description of Additional Supplementary Files

**File name:** Supplementary Data 1
**Description:** Quality control report for LCMS data obtained at positive ion mode.

**File name:** Supplementary Data 2
**Description:** Quality control report for LCMS data obtained at negative ion mode.
