# Supplementary material for: Spatial Metabolomics Reveals the Multifaceted Nature of Lamprey Buccal Gland and Its Diverse Mechanisms for Blood-Feeding
Source: Commun Biol. 2023 Aug 28;6:881. doi: 10.1038/s42003-023-05250-x (PMC10462737; doi:10.1038/s42003-023-05250-x)
Supplement: Supplementary file 3 — Supplementary Data 1 [file 42003_2023_5250_MOESM3_ESM.pdf]

1 Introduction
2 Chromatogram
3 MS1
4 MS2
5 Reference

RawHumms Data Quality Control Report

04, September, 2022

1 Introduction

Robust and reproducible data is essential to ensure confidence in analytical results and is particularly important for large-scale metabolomics studies. Therefore raw data need to be inspected before data processing and statistical analysis in order to detect measurement bias and verify system consistency. In liquid chromatography mass spectrometry (LCMS) based metabolomics studies, proper quality control (QC) checks are particularly important to ensure reliable and comparable results within experimental measurements [1-2].

RawHummus is an user-friendly web application for rapid data quality check based on raw QC samples. It generates an HTML report with interactive plots, statistics and illustrations that help users evaluate their data quality and LCMS system performance.

2 Chromatogram

2.1 TIC plot

`Total Ion Current (TIC)` chromatogram represents the summed ion intensity across each scan in the analysis. **The interactive overlaid TIC plot** can be used for rapid inspection of retention time (RT) and ion intensity fluctuations.

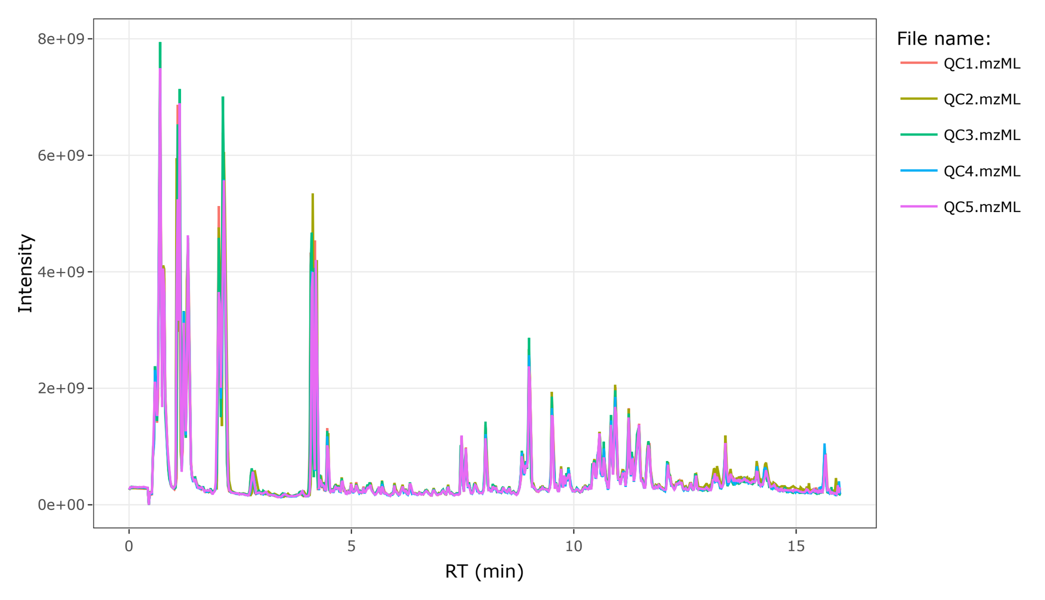

2.2 Summed TIC Plot

Summed TIC plot is another quick-and-dirty way to overview global ion intensity variations among QC samples. It summed TIC across the entire points (scans) from the analysis. Dashed red line is **mean** of summed TIC and blue lines represent **mean + 2SD** and **mean - 2SD**, respectively.

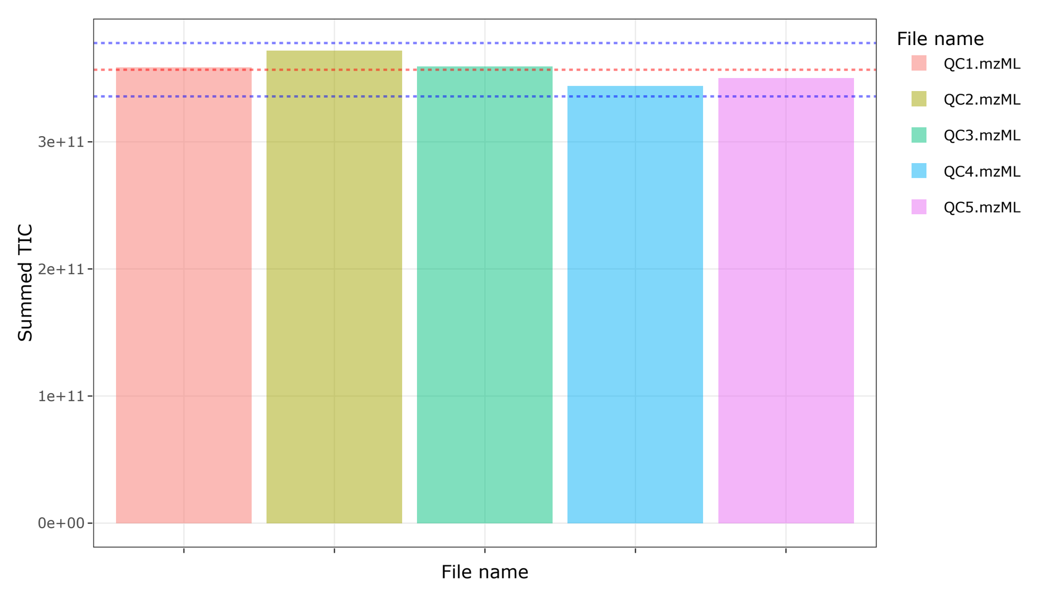

2.3 TIC Correlation Analysis

`Pearson correlation` is used to quantify the metabolic profile similarity among QC samples. Pearson correlation coefficient (**R**) over **0.85** indicate high metabolic profile similarity in RT and chromatogram peak shape. If the value is below 0.85, it will be highlight in red in Table 1.

> **Note that** RT were binned by 0.1 min for Pearson correlation calculation.

Table 1: Metabolic profile similarity

	QC1.mzML	QC2.mzML	QC3.mzML	QC4.mzML	QC5.mzML
QC1.mzML	1	0.967	0.995	0.982	0.981
QC2.mzML	0.967	1	0.973	0.992	0.992
QC3.mzML	0.995	0.973	1	0.985	0.988
QC4.mzML	0.982	0.992	0.985	1	0.997
QC5.mzML	0.981	0.992	0.988	0.997	1

> **Note that** `TIC Correlation Analysis` is mainly used to evaluate peak shape similarity and RT consistency. QC files with ion intensity drift but similar profiles could still have good Pearson correlation coefficient.

3 MS1

3.1 Auto Peaks Evaluation

In order to accurately monitor variations in mass, RT and ion intensity, RawHummus automatically selects **6** peaks across the entire RT range, and use them to evaluate LCMS system.

Below are the Extracted ion chromatogram (EIC) of the 6 selected ions. You can interactively view, inspect and compare them among different QC samples.

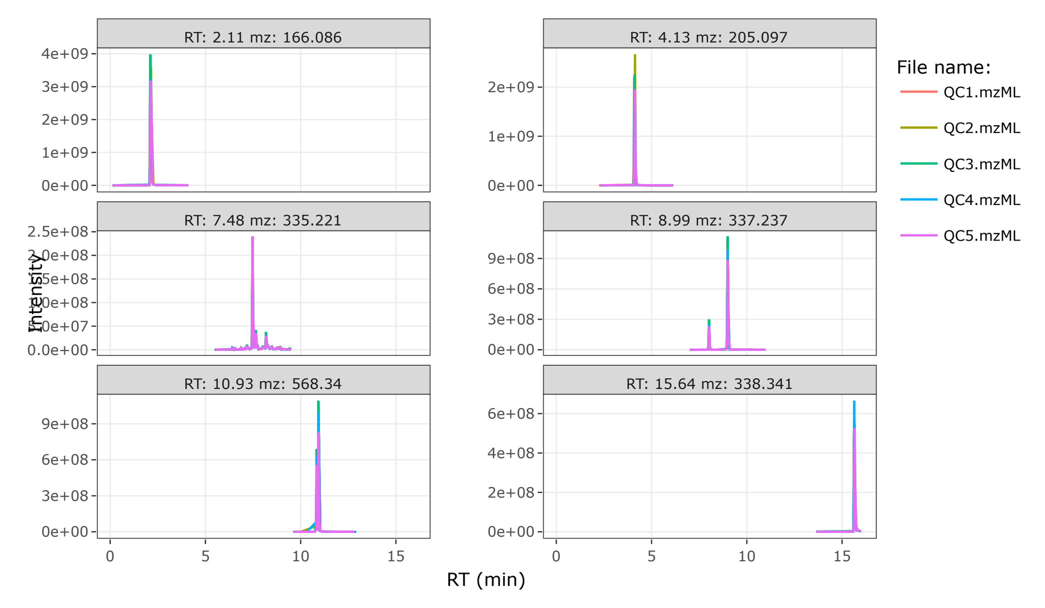

RawHummus performs a simple statistics to make rapid evaluation. The table below summarized the comparison result, in which maximum RT difference, mass difference and ion intensity difference, and Intensity CV are given.

`Max RT Diff (min)`: is the maximum retention time variation (in min unit). Small value indicates a good retention time consistency. If the maximum retention time variation is over 1 min, the value will be highlight in red in Table 2.

`Max Mass Diff (ppm)`: is the maximum mass variation (in ppm unit). Small values indicate good mass accuracy. If the maximum mass variation is over 5 ppm, the value will be highlight in red in Table 2.

`Max Intensity Fold Change`: is the maximum ion intensity variation. The value closer to 1 suggests that the ion intensity is stable. If the maximum intensity ratio is over 2, the value will be highlight in red in Table 2.

If a peak is missing in some of your samples, NA values will be give in the table. You need to carefully inspect the peak so as to evaluate the reproducibility.

`Intensity CV (%)`: is the ion intensity coefficient of variation (also termed as relative standard deviation, STD). Smaller value indicates better ion intensity consistency. If the intensity CV is over 30%, the value will be highlight in red in Table 2.

Table 2: Summary of auto-selected peaks

Peak	Max RT Diff (min)	Max Mass Diff (ppm)	Max Intensity Ratio	Intensity CV (%)
RT: 2.11 mz: 166.086	0.02	0.92	1.25	10
RT: 4.13 mz: 205.097	0.05	0.67	1.37	13.44
RT: 7.48 mz: 335.221	0	0.73	1.14	5.79
RT: 8.99 mz: 337.237	0	0.81	1.27	8.66
RT: 10.93 mz: 568.34	0.02	0.75	1.32	10.44
RT: 15.64 mz: 338.341	0.03	0.81	1.47	15.06

3.2 User defined peaks

Additionally, users could add their peaks of interests for inspection and comparison. If these peaks are defined in RawHummus and are found in the data. Similar plots and a data summary table will be given below. Otherwise, this section will be left blank.

> **Note that** noise peaks can be used to minitor the mass accuracy variation, but they may not work well to evalute RT and ion intensity variation.

```
## [1] "User defined peaks are not found"
```

4 MS2

MS/MS fragmentation is important for metabolite identification. RawHummus is also able to identify problems with regard to fragmentation.

> **Note that** if your data files do not contain any MS/MS information, this section will be left blank.

4.1 Number of MS2 Events

`Number of triggered MS/MS`: is the total number of MS/MS events in the sample. Similar number of triggered MS/MS events indicates good reproducibility.

Table 4: Summary of MS2 events

filename	MS2 Events
QC1.mzML	5493
QC2.mzML	5494
QC3.mzML	5497
QC4.mzML	5495
QC5.mzML	5496

4.2 Precursor Distribution across Mass Range

`Precursor Distribution across Mass` plot is used to visualize the mass range proportion of fragmented precursors.

- It can be used to check peaks at which mass ranges are mainly fragmented.
- High similarity in `Precursor Distribution across Mass` among QC samples indicates good reproducibility in MS2.

```
## $`1`
```

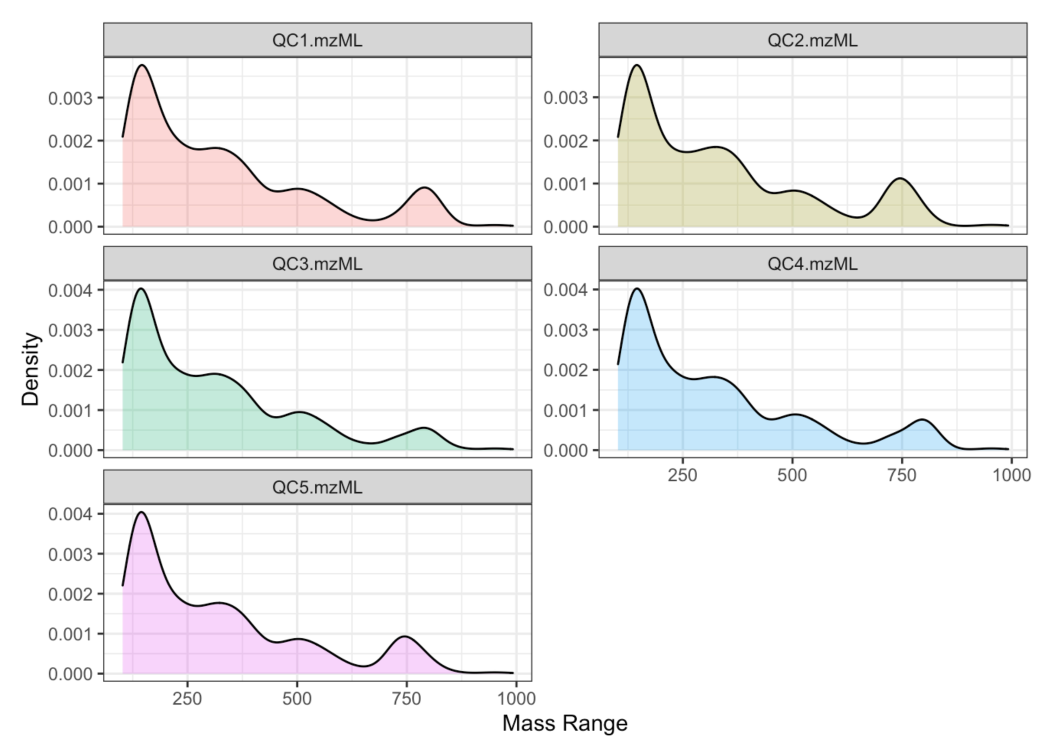

`Cosine Similarity` is used to quantify the similarity of precursor distribution across mass range plots among QC samples. Cosine similarity over 0.85 indicate high similarity. If the value is below 0.85, it will be highlight in red in Table 5.

> **Note that** precursor ions were binned by 10 Da for Pearson correlation calculation.

Table 5: Precursor Distribution across mass similarity

	QC1.mzML	QC2.mzML	QC3.mzML	QC4.mzML	QC5.mzML
QC1.mzML	1	0.965	0.991	0.981	0.969
QC2.mzML	0.965	1	0.973	0.967	0.981
QC3.mzML	0.991	0.973	1	0.987	0.978
QC4.mzML	0.981	0.967	0.987	1	0.973
QC5.mzML	0.969	0.981	0.978	0.973	1

4.3 Precursor Distribution across RT

`Precursor Distribution across RT` plot is used to visualize the number of fragmented precursors at each RT (or scan). It can be useful to spot any signal dropouts during data acquisition.

- In both **data-dependent acquisition (DDA)** and **data-independent acquisition (DIA)** mode, you are expected to see a continuous precursor ions distribution across the entire RT range.
- High similarity in `Precursor Distribution across RT` among QC samples indicates good reproducibility in MS2.

```
## $`1`
```

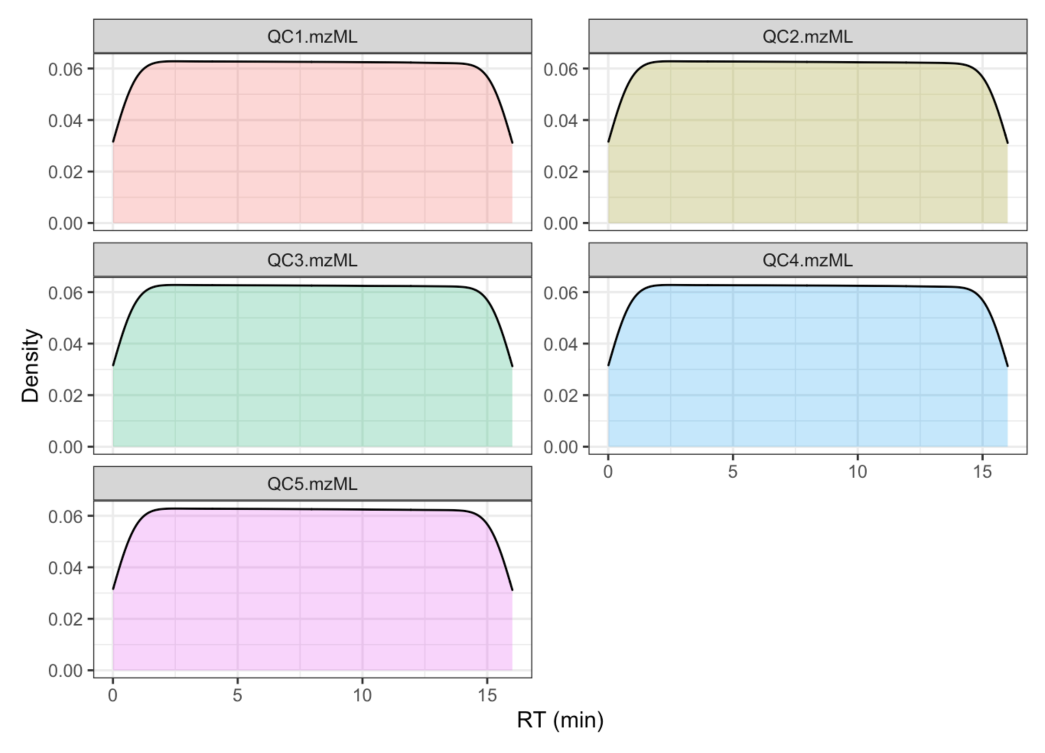

`Cosine Similarity` is used to quantify the similarity of precursor distribution across RT plots among QC samples. Cosine similarity over 0.85 indicate high similarity. If the value is below 0.85, it will be highlight in red in Table 6.

> **Note that** RT were binned by 0.05 min for Pearson correlation calculation.

Table 6: Precursor Distribution across RT similarity

	QC1.mzML	QC2.mzML	QC3.mzML	QC4.mzML	QC5.mzML
QC1.mzML	1	0.999	0.999	0.999	0.999
QC2.mzML	0.999	1	0.999	0.999	0.999
QC3.mzML	0.999	0.999	1	0.999	0.999
QC4.mzML	0.999	0.999	0.999	1	0.999
QC5.mzML	0.999	0.999	0.999	0.999	1

5 Reference

[1] Scalbert, A., Brennan, L., Fiehn, O., Hankemeier, T., Kristal, B.S., van Ommen, B., Pujos-Guillot, E., Verheij, E., Wishart, D. and Wopereis, S., 2009. Mass-spectrometry-based metabolomics: limitations and recommendations for future progress with particular focus on nutrition research. Metabolomics, 5(4), pp.435-458.

[2] Begou, O., Gika, H.G., Theodoridis, G.A. and Wilson, I.D., 2018. Quality Control and Validation Issues in LC-MS Metabolomics. Methods in molecular biology (Clifton, NJ), 1738, pp.15-26.
